# Supplementary material for: Blurring the boundary between homogenous and heterogeneous catalysis using palladium nanoclusters with dynamic surfaces
Source: Nat Commun. 2021 Aug 17;12:4965. doi: 10.1038/s41467-021-25263-6 (PMC8371125; doi:10.1038/s41467-021-25263-6)
Supplement: Supplementary file 1 — Supplementary Information [file 41467_2021_25263_MOESM1_ESM.pdf]

SUPPORTING INFORMATION

Blurring the Boundary between Homogenous and Heterogeneous Catalysis using Palladium Nanoclusters with Dynamic Surfaces

Israel Cano,[1] Andreas Weilhard,[1] Carmen Martin,[1] Jose Pinto,[1,2] Rhys W. Lodge,[1] Ana R. Santos,[2] Graham A. Rance,[1,3] Elina Harriet Åhlgren,[1] Erlendur Jónsson,[4,5] Jun Yuan,[6] Ziyou Y. Li,[7] Peter Licence,[2] Andrei N. Khlobystov[1] and Jesum Alves Fernandes[1*]

[1]School of Chemistry, University of Nottingham, Nottingham, NG7 2RD, UK

[2]GSK Carbon Neutral Laboratories for Sustainable Chemistry, University of Nottingham, Nottingham NG7 2TU, UK

[3]Nanoscale and Microscale Research Centre, University of Nottingham, Nottingham NG7 2RD, UK

[4]Department of Chemistry, University of Cambridge, Cambridge CB2 1EW, UK

[5]Department of Physics, Chalmers Technical University, Gothenburg 412 96, SE

[6]Department of Physics, University of York, York YO10 5DD, UK

[7]Nanoscale Physics Research Laboratory, School of Physics and Astronomy, University of Birmingham, Birmingham B15 2TT, UK

*jesum.alvesfernandes@nottingham.ac.uk

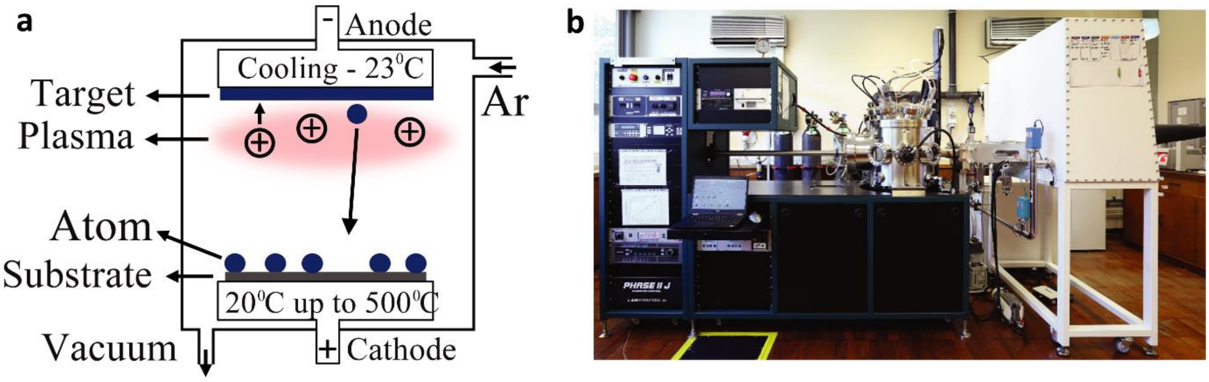

Supplementary Figure 1 Method for Pd nanoclusters preparation. a Schematic illustration of magnetron sputtering process. **b** Photo of the magnetron sputtering system used in this work located at the School of Chemistry – University of Nottingham.

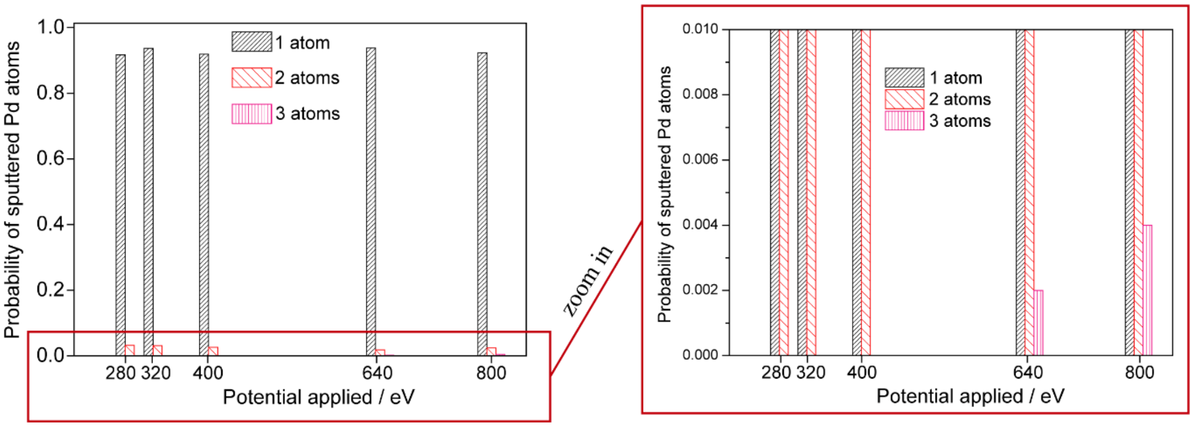

Supplementary Figure 2 Optimisation of potential applied for magnetron sputtering Pd depositions. Probability of number of Pd atoms sputtered depending on potential applied calculated using computational methods.

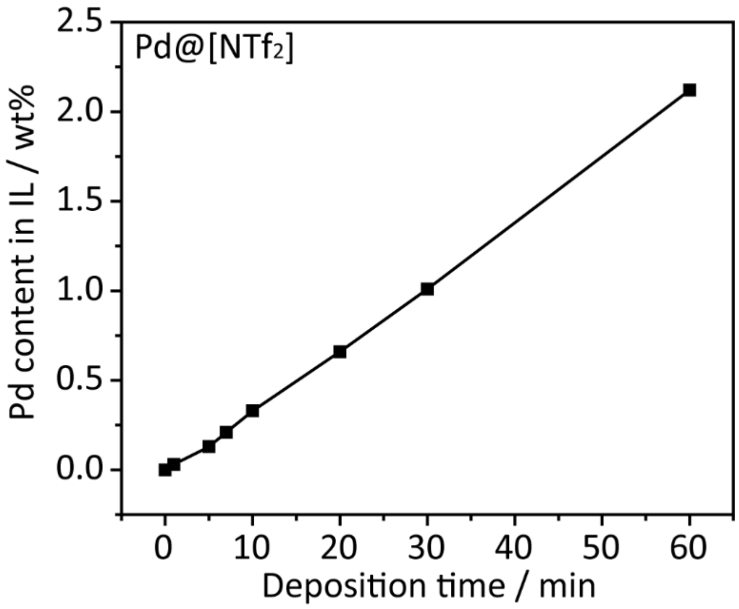

Supplementary Figure 3 Different deposition time of Pd species in [C_4C_1Im][NTf_2]. Pd species deposition in [C_4C_1Im][NTf_2] shows a linear increase in the Pd concentration as the deposition time is increased.

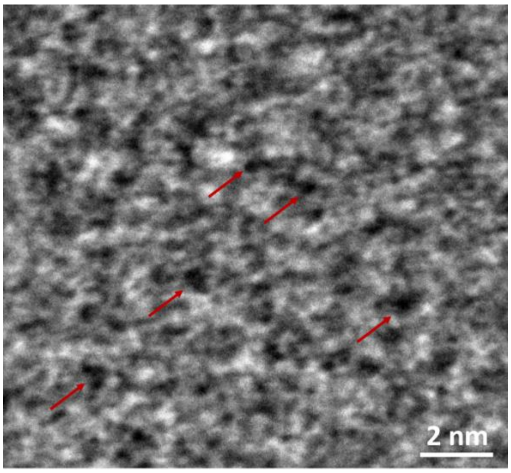

Supplementary Figure 4 TEM image of 1Pd@[NTf$_2$]. No Pd nanocluster with mean diameter size above 1 nm was observed. The arrows indicate the possible presence of single Pd atoms and nanoclusters with mean diameter size below 1 nm.

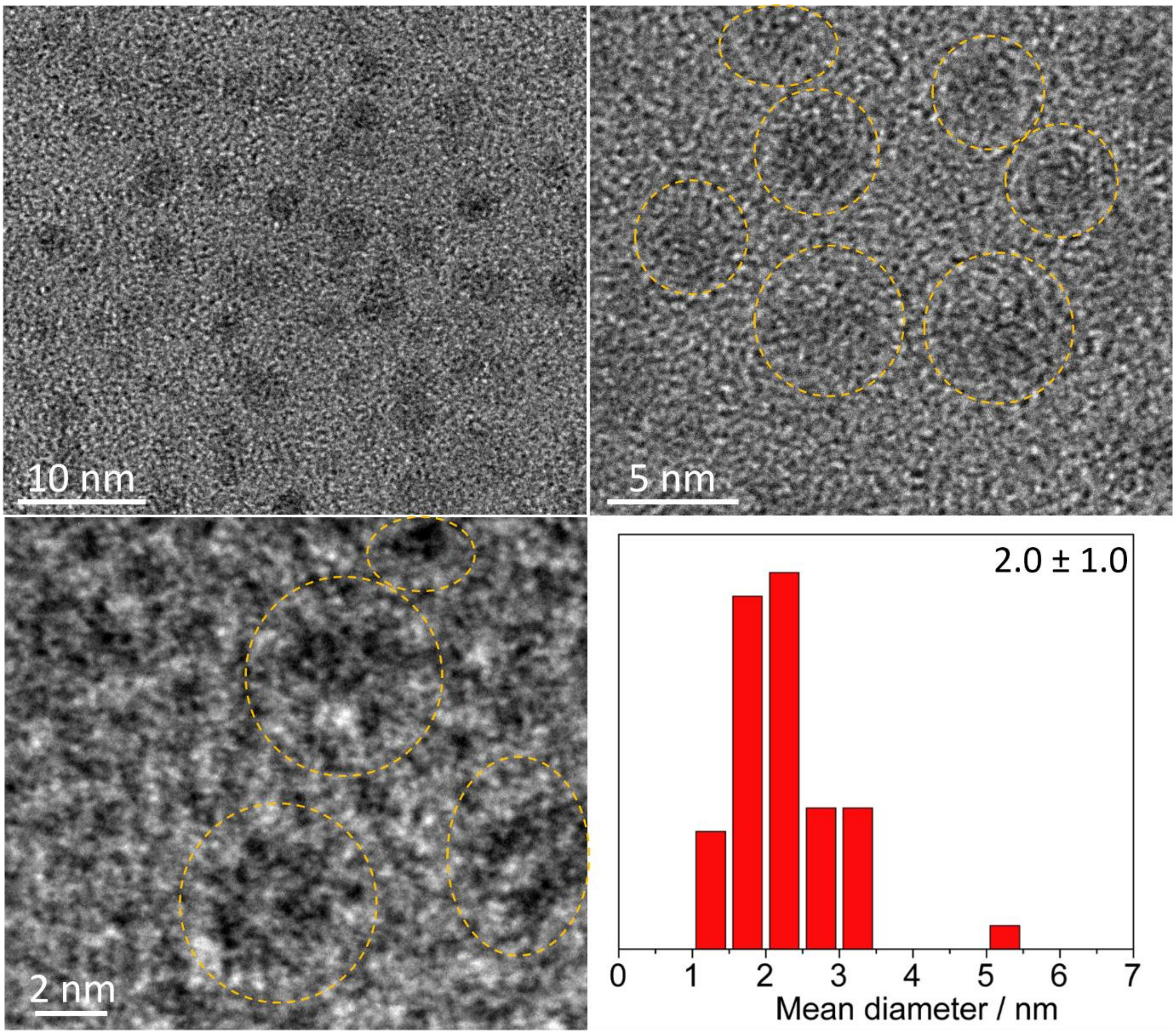

Supplementary Figure 5 TEM images and particle size distribution histogram of 5Pd@[NTf$_2$]. The large size variation is associated to the dynamic shell surrounding the Pd core.

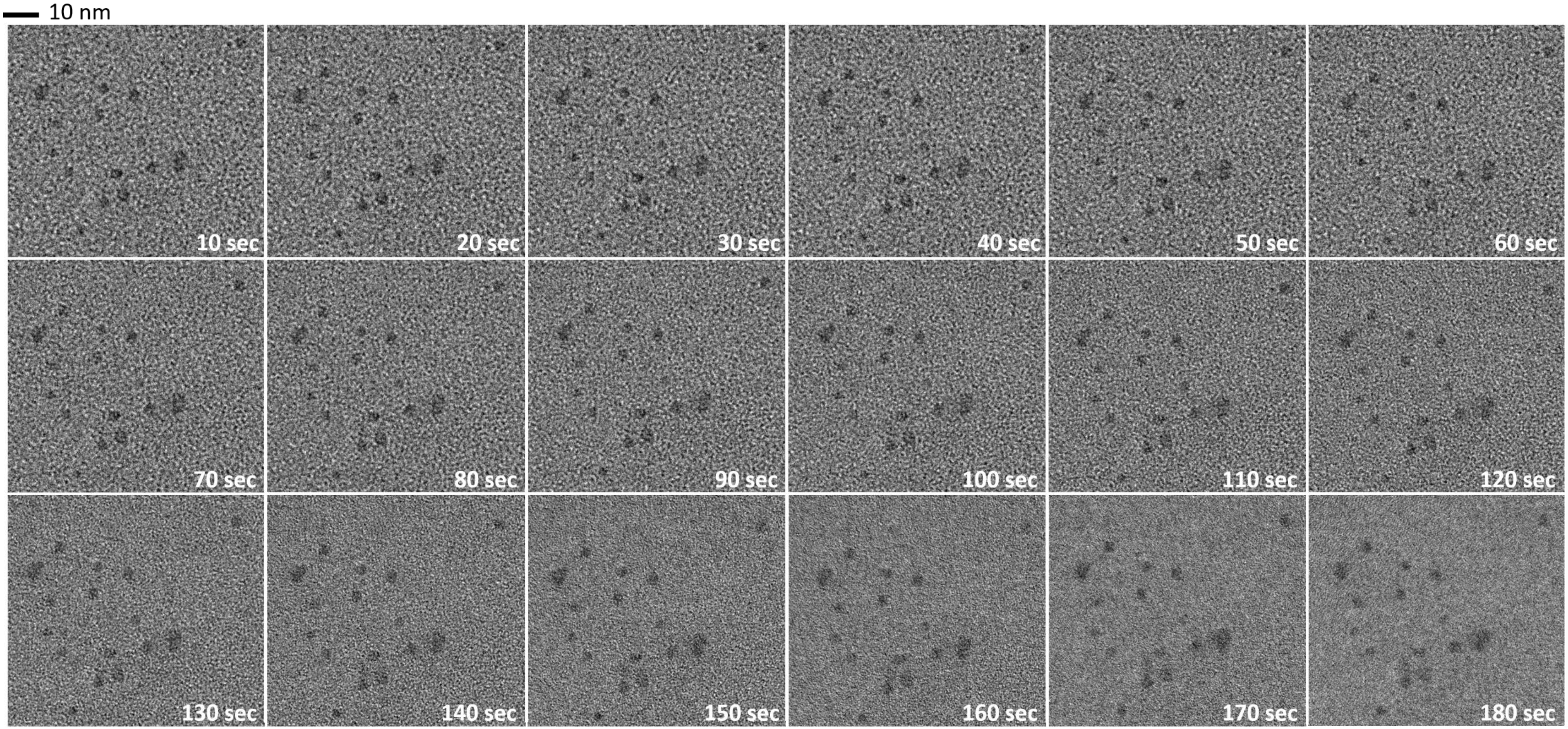

Supplementary Figure 6 TEM images acquired time series of 5Pd@[NTf$_2$] using same temperature as the macroscopic reaction, which is the closest set of conditions to the real reaction possible for atomic scale imaging. This detailed sequence of TEM images demonstrates the dynamic nature of the nanoclusters. The Pd nanoclusters exhibited significant dynamics in the structure at the atomic level in individual nanoclusters, while the overall integrity of the nanoclusters was maintained.

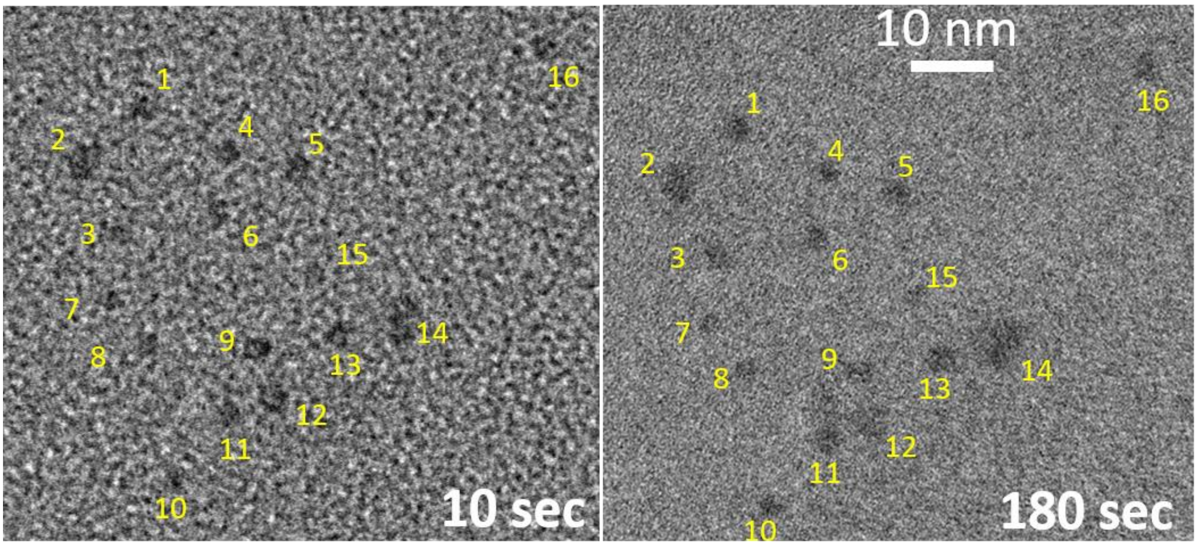

Supplementary Figure 7 TEM images acquired over time of 5Pd@[NTf_2]. These TEM images clearly demonstrate that there is no electron beam damage over Pd clusters, and therefore rules out the hypotheses of Pd dynamic nanoclusters being formed under electron irradiation.

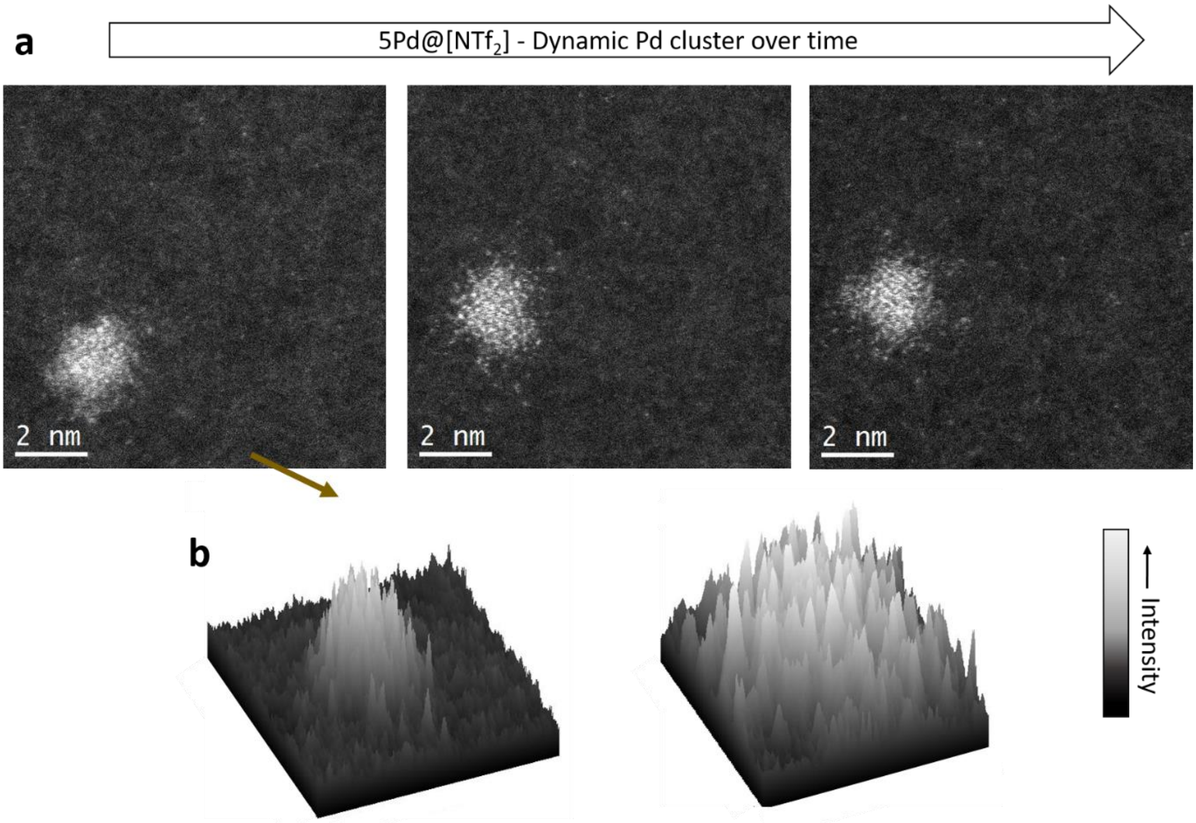

Supplementary Figure 8 AC-STEM images of 5Pd@[NTf_2] sample over time. a from left to right: 30, 90 and 150 sec. **b** The 3D histograms were taken from image **a** to highlight the Pd nanoclusters surfaces, left is a 3D histogram of the entire image and, right is a 3D histogram taken only from the Pd nanocluster. The intensity bar refers to AC-STEM image contrast which correlates with the number of Pd atoms in the image.

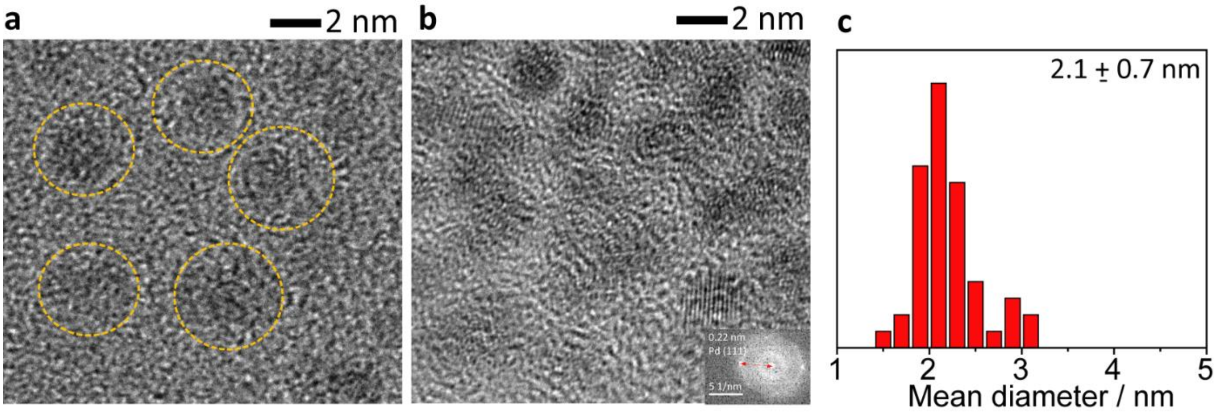

Supplementary Figure 9 TEM images of 7Pd@[NTf$_2$] show two distinct Pd clusters morphologies. a Pd dynamic clusters and **b** Pd compact cluster. **c** Particle size distribution for 7Pd@[NTf$_2$].

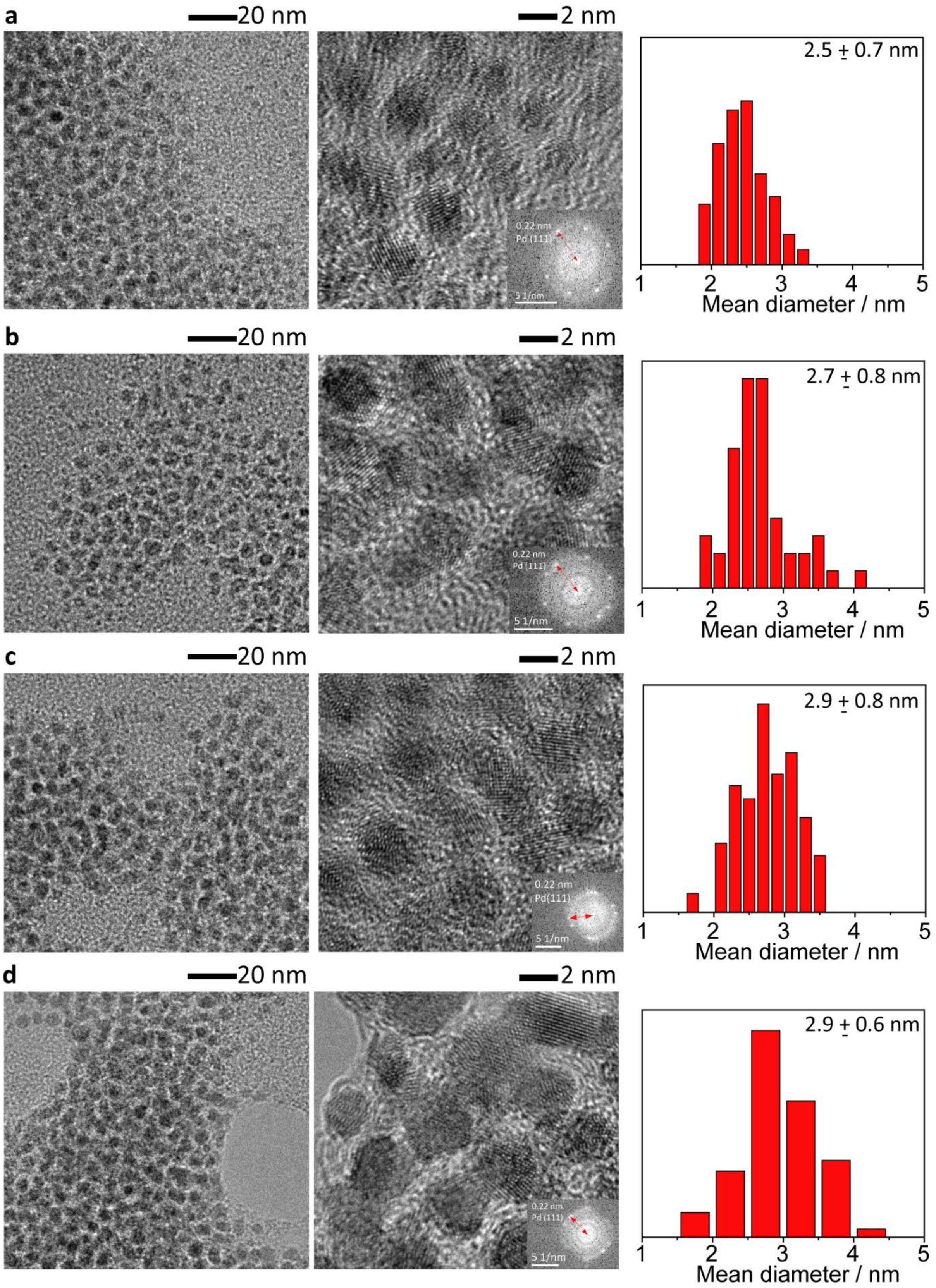

Supplementary Figure 10 TEM images and particle size distribution histograms of samples. a 10Pd@[NTf_2]; **b** 20Pd@[NTf_2], **c** 30Pd@[NTf_2] and **d** 60Pd@[NTf_2].

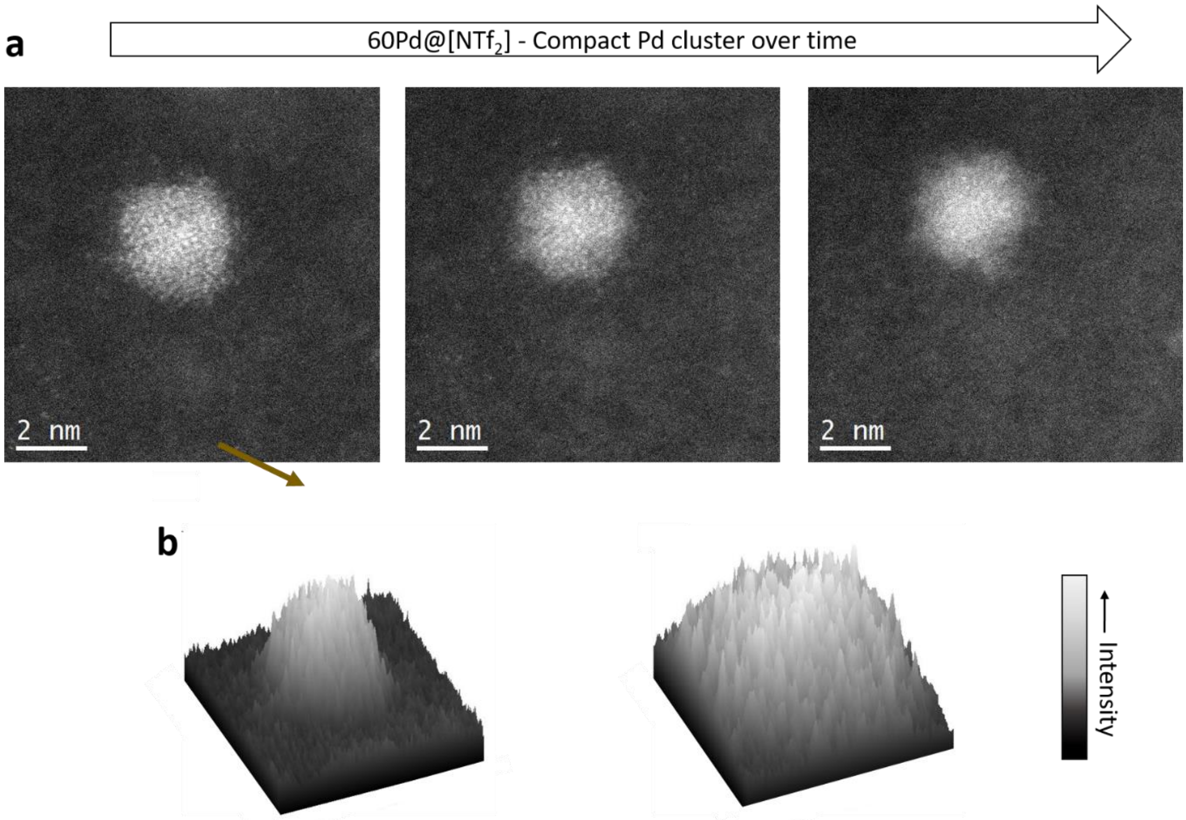

Supplementary Figure 11 AC-STEM images of 60Pd@[NTf_2] sample over time. a from left to right: 30, 90 and 150 sec. **b** The 3D histograms were taken from image **a** to highlight the Pd nanoclusters surfaces, left is a 3D histogram of the entire image and, right is a 3D histogram taken only from the Pd nanocluster. The intensity bar refers to AC-STEM image contrast which correlates with the number of Pd atoms in the image

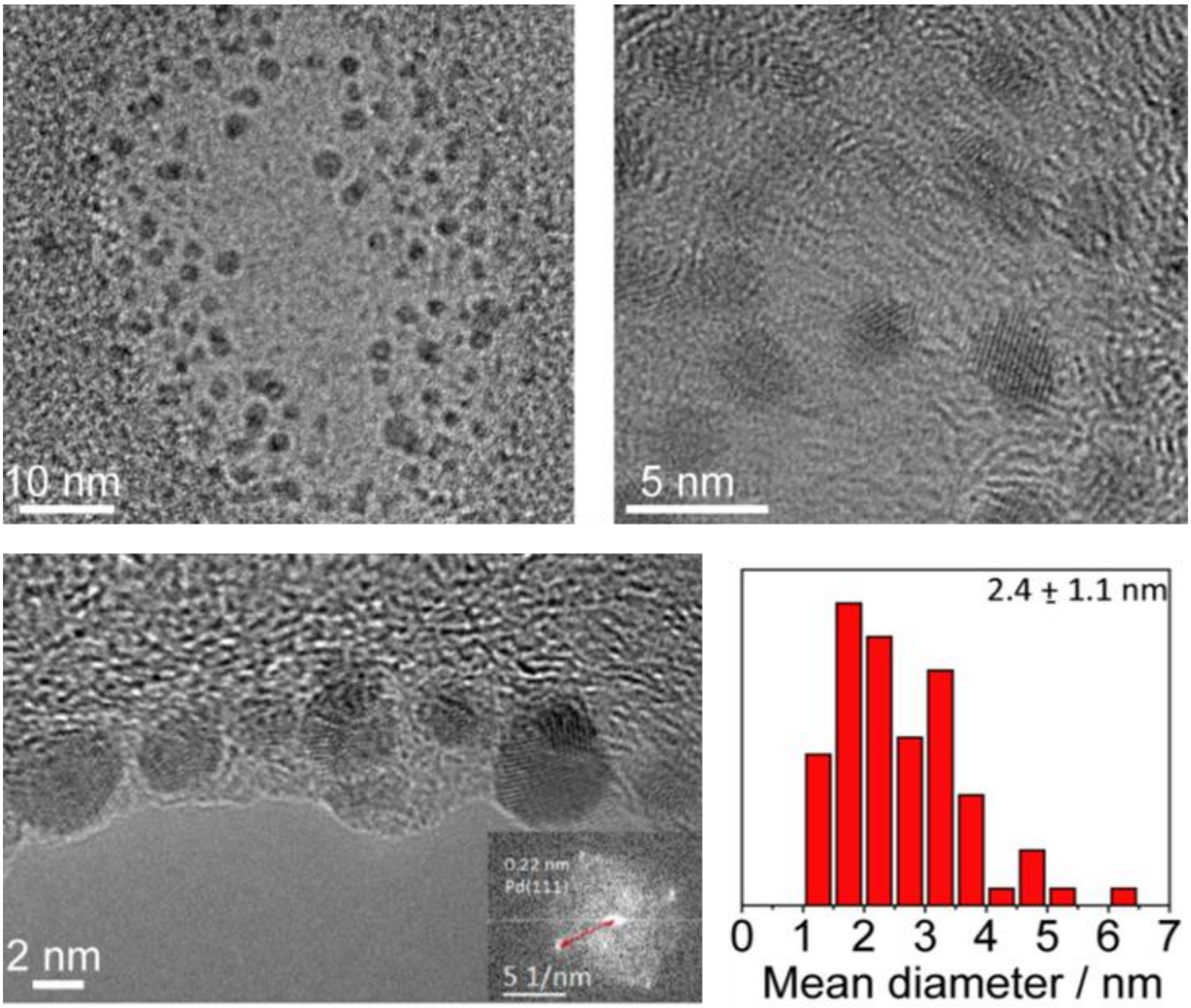

Supplementary Figure 12 TEM images and particle size distribution histograms of 5Pd@[PF_6]. TEM images of 5Pd@[PF_6] shows nanocluster with a compact structure, with metallic Pd lattice planes being observed.

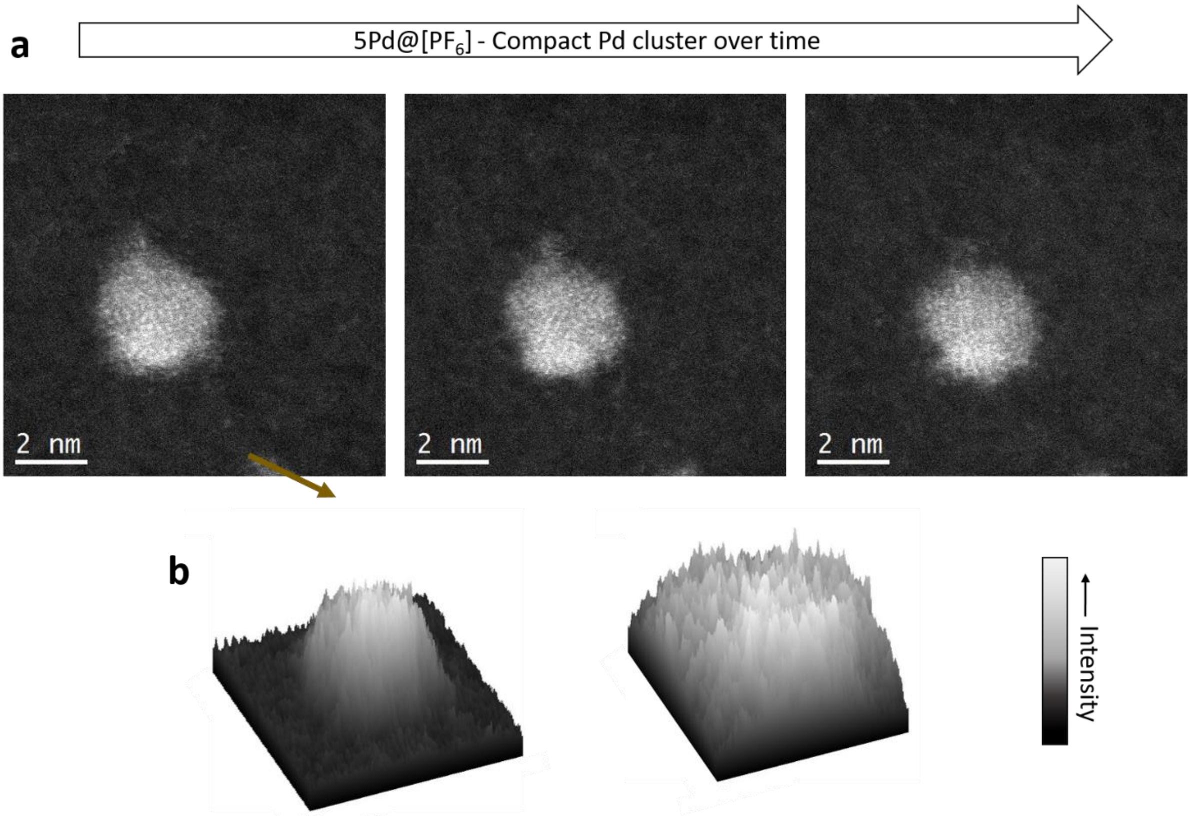

Supplementary Figure 13 AC-STEM images of 5Pd@[PF_6] sample over time. a from left to right: 30, 90 and 150 sec. **b** The 3D histograms were taken from image **a** to highlight the Pd nanoclusters surfaces, left is a 3D histogram of the entire image and, right is a 3D histogram taken only from the Pd nanocluster. The intensity bar refers to AC-STEM image contrast which correlates with the number of Pd atoms in the image.

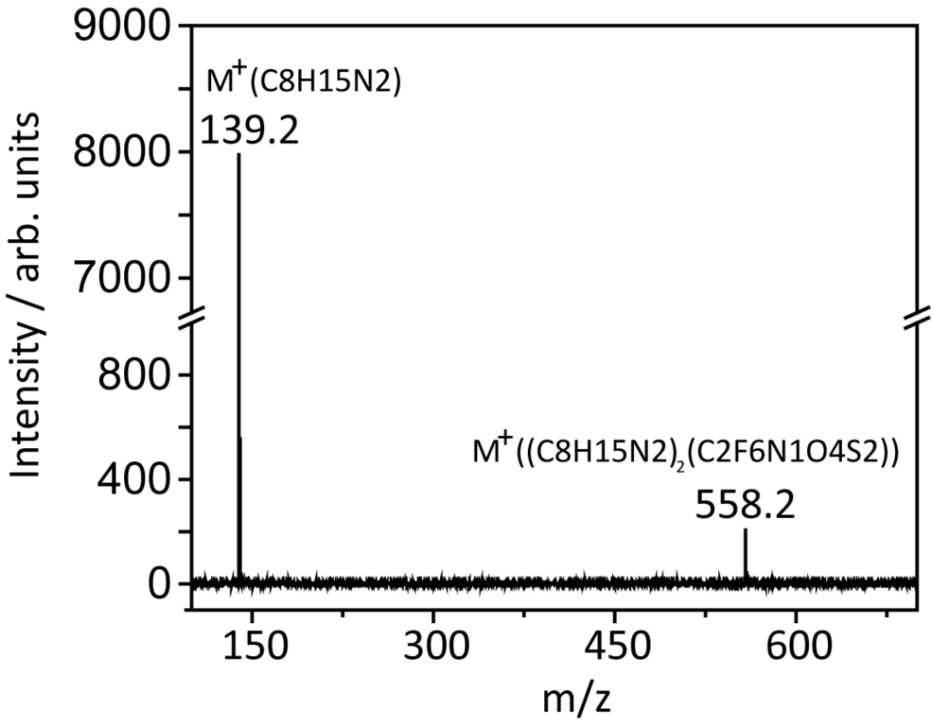

Supplementary Figure 14 [C_4C_1Im][NTf_2] wide scan mass spectrum. A good consistency between positive mode ESI-MS and MALDI spectra was observed.

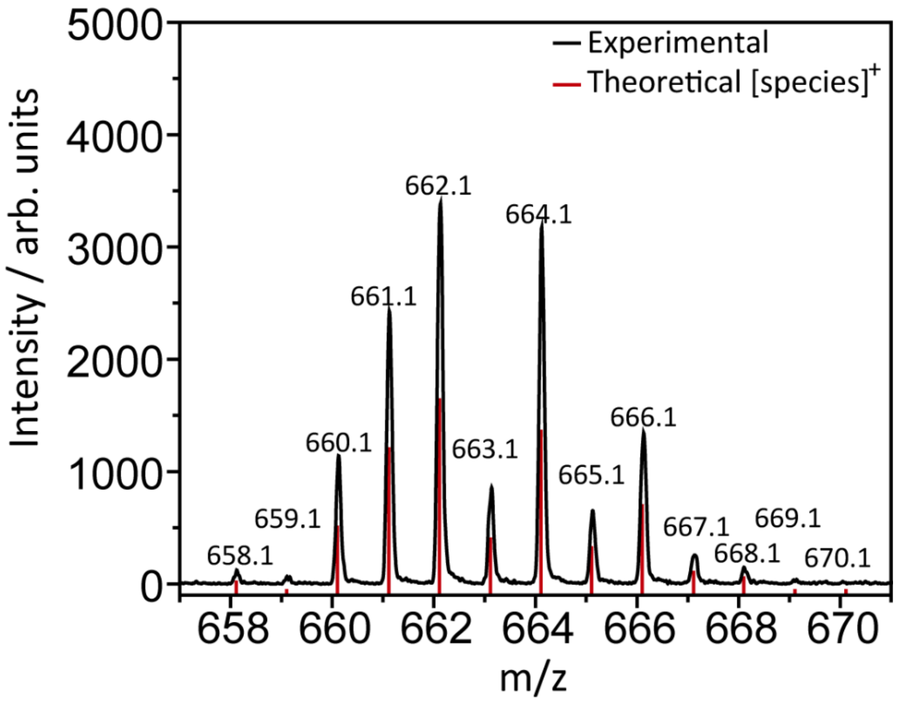

Supplementary Figure 15 MALDI-TOF mass spectrum of 1Pd@[NTf_2] and 5Pd@[NTf_2]. The presence of single Pd atoms was observed for these samples. Theoretical [species]$^+$ (Pd(C8H14N2)$_2$(C2NO4S2)).

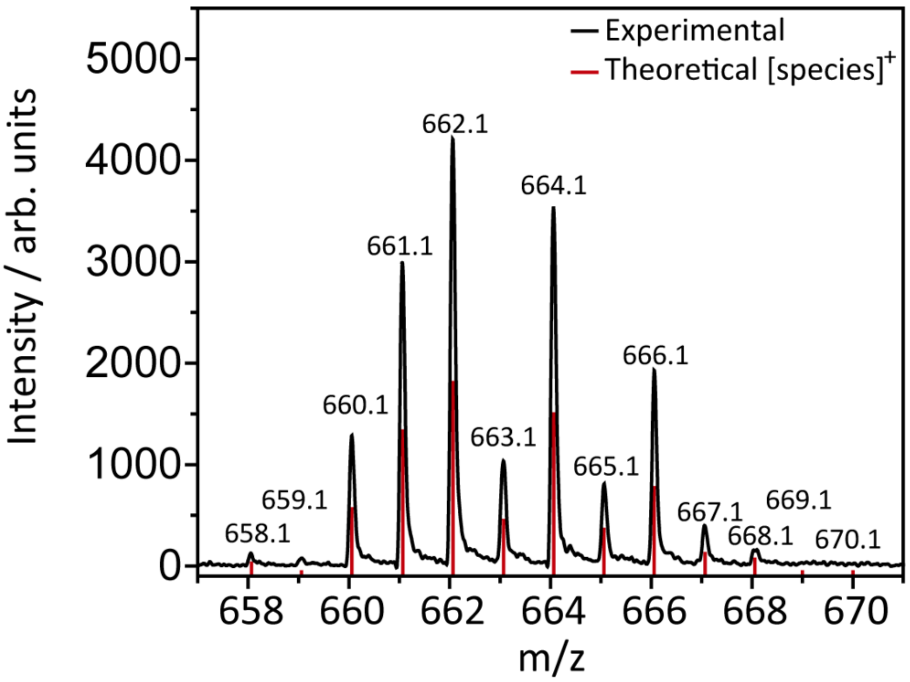

Supplementary Figure 16 MALDI-TOF mass spectrum confirmed the presence of single Pd atoms for samples 10Pd@[NTf$_2$]. Theoretical [species]$^+$ (Pd(C8H14N2)$_2$(C2NO4S2)).

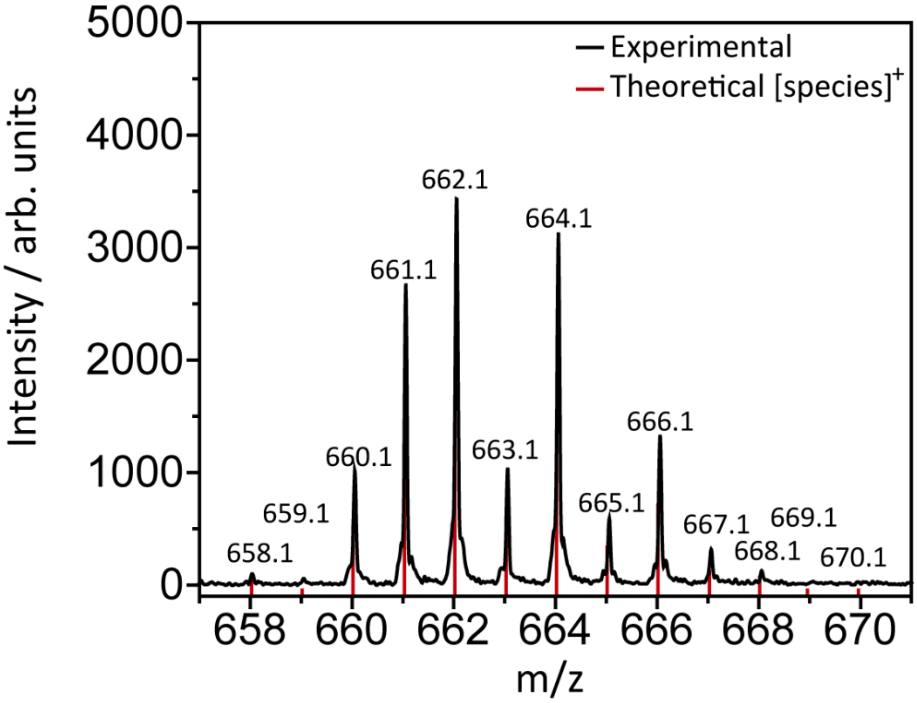

Supplementary Figure 17 MALDI-TOF mass spectrum of 30Pd@[NTf$_2$]. The presence of single Pd atoms was observed for this sample. Theoretical [species]$^+$ (Pd(C8H14N2)$_2$(C2NO4S2)).

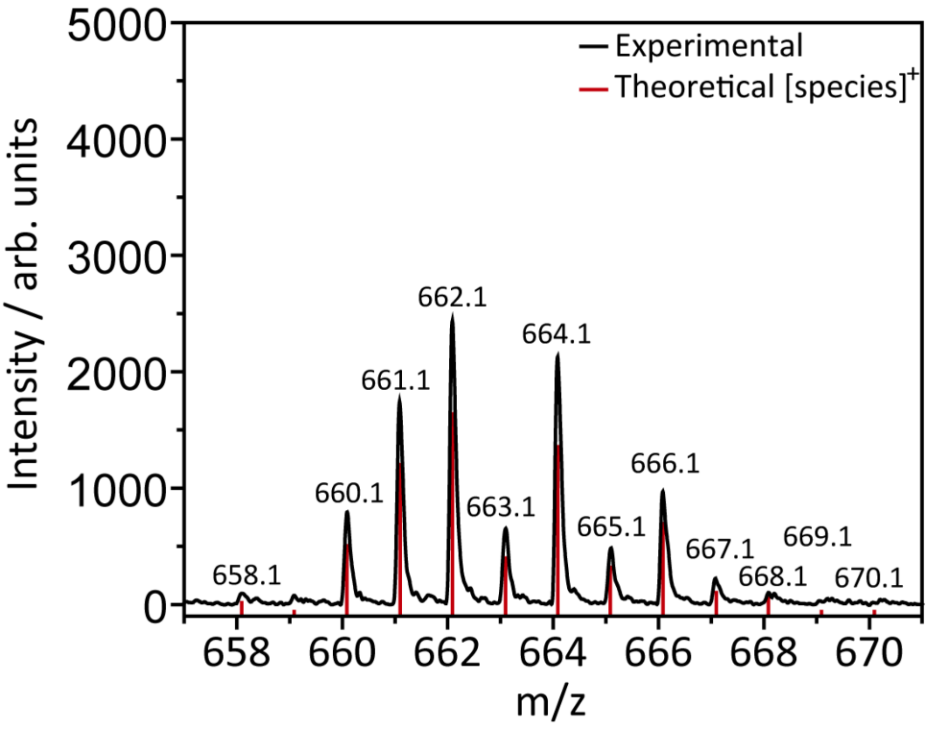

Supplementary Figure 18 MALDI-TOF mass spectrum of the 60Pd@[NTf$_2$]. The presence of single Pd atoms was observed for this sample. Theoretical [species]$^+$ (Pd(C8H14N2)$_2$(C2NO4S2)).

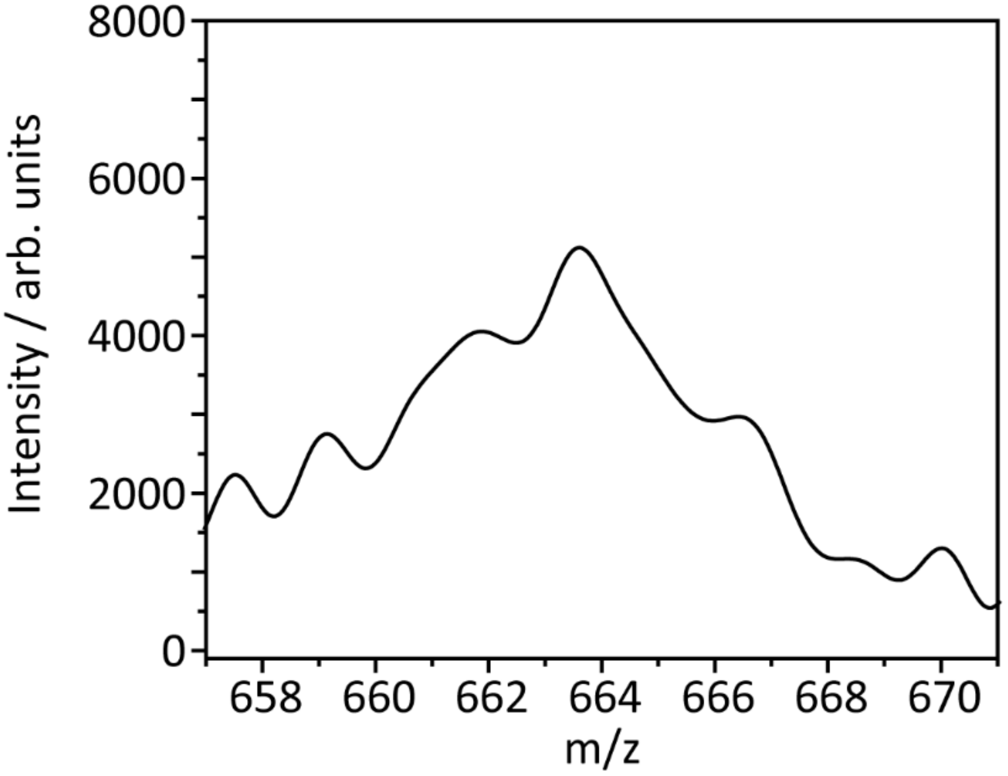

Supplementary Figure 19 MALDI-TOF of the 5Pd@[PF$_6$]. No presence of single Pd atoms was detected for this sample.

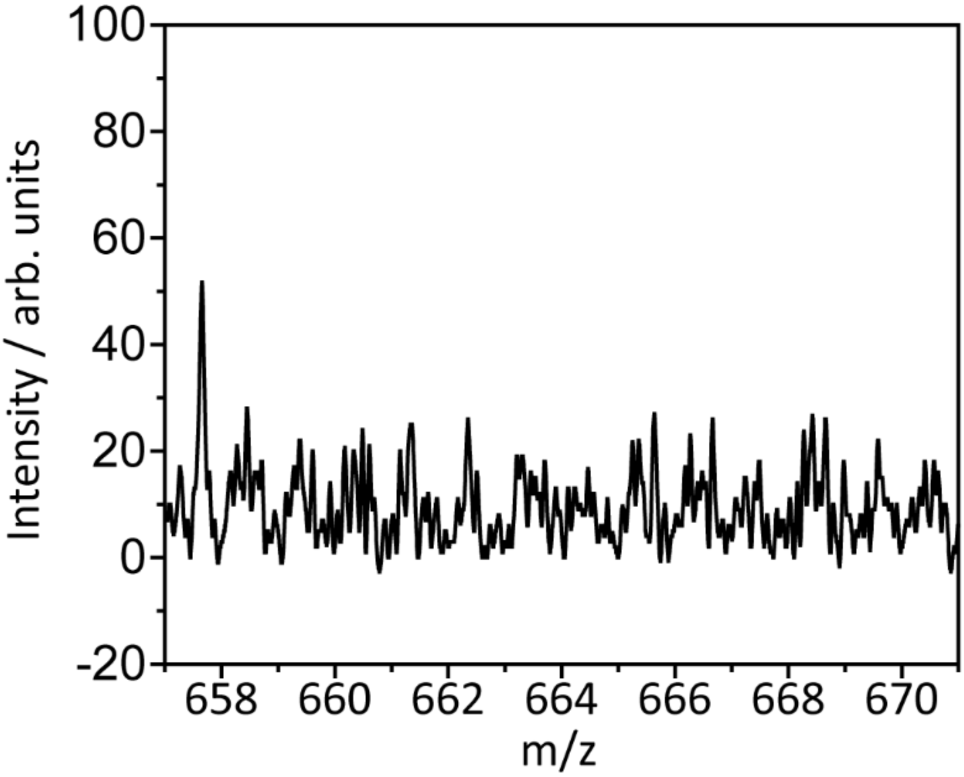

Supplementary Figure 20 MALDI-TOF of the 30Pd@[$C_4C_1C_1$Im][NTf_2]. No presence of single Pd atoms was detected for this sample.

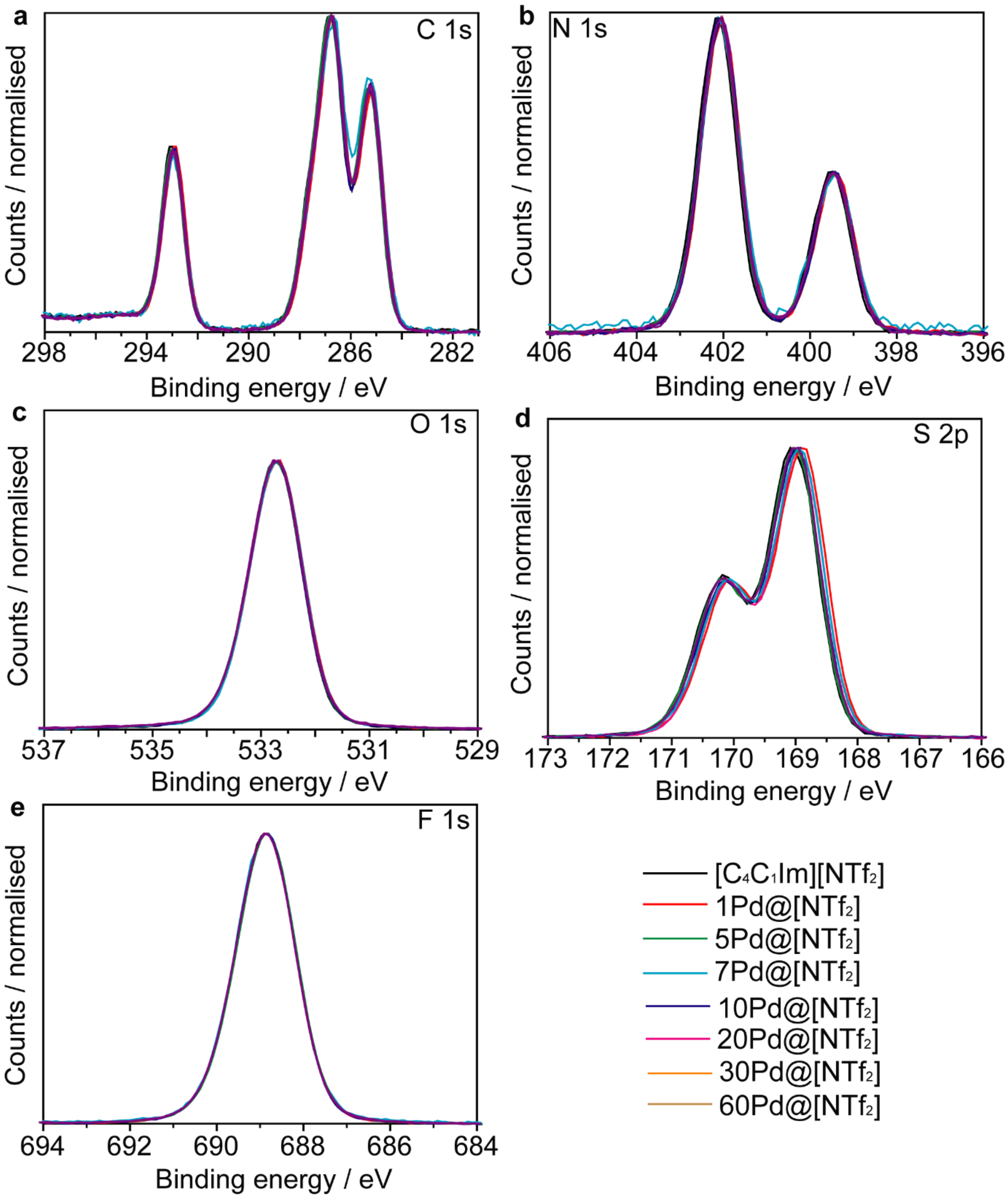

Supplementary Figure 21 HR-XPS measurements of pure IL and Pd@IL system with different Pd contents. **a** C1s, **b** N1s, **c** O1s, **d** S2p and **e** F1s. After deposition of Pd NPs, no significant changes were observed by XPS. The peak shape, FWHM, BEs or composition of the IL components appeared to be unaffected by the presence of Pd nanoclusters due to large excess of IL over Pd nanoclusters.

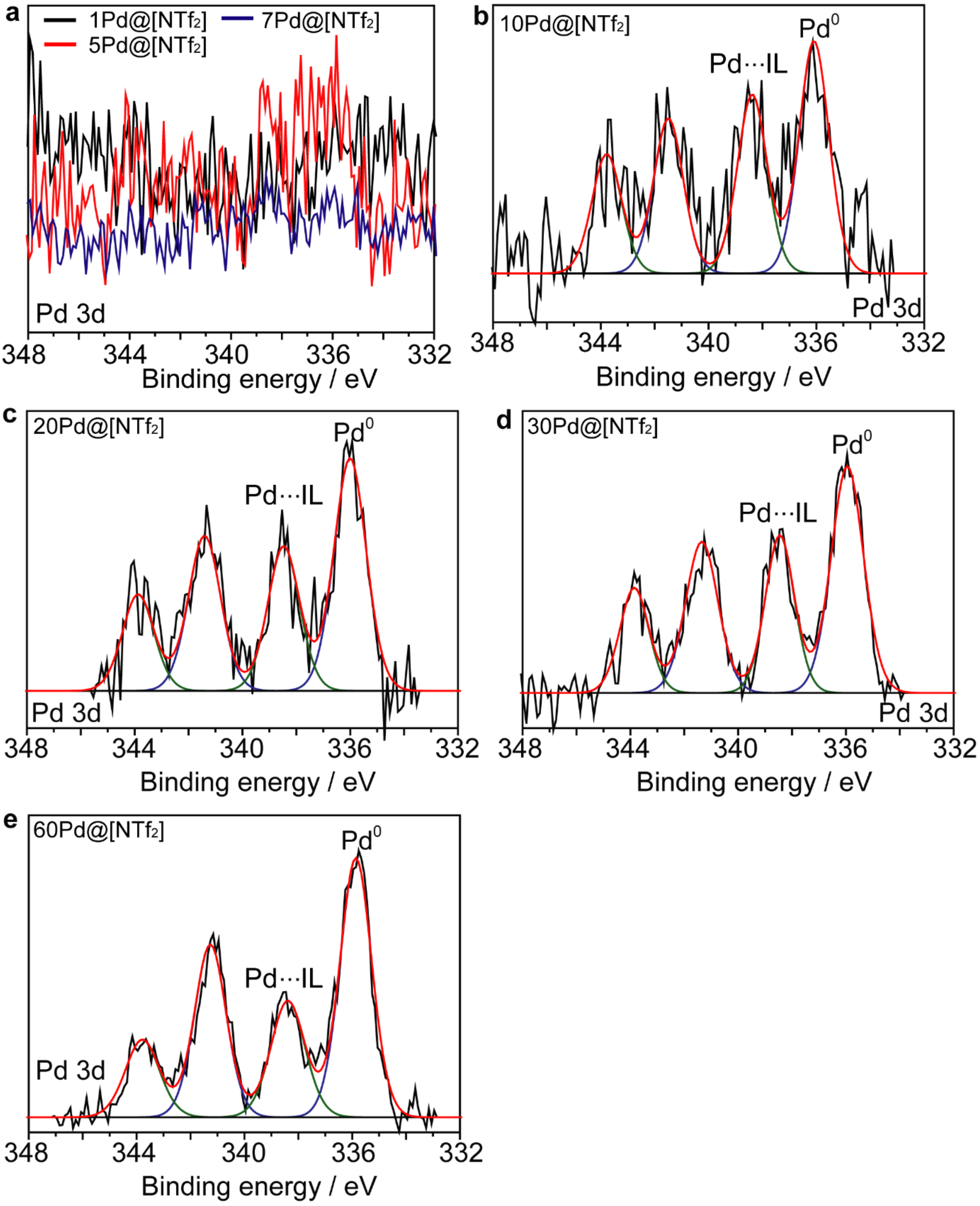

Supplementary Figure 22 HR-XPS measurements of Pd deposited in [C_4C_1Im][NTf_2] with different Pd concentrations. **a** 0.03, 0.12 and 0.19 wt%; **b** 0.33 wt%; **c** 0.66 wt%; **d** 1.01 wt%; **e** 2.12 wt%. The black line represents the experimental data.

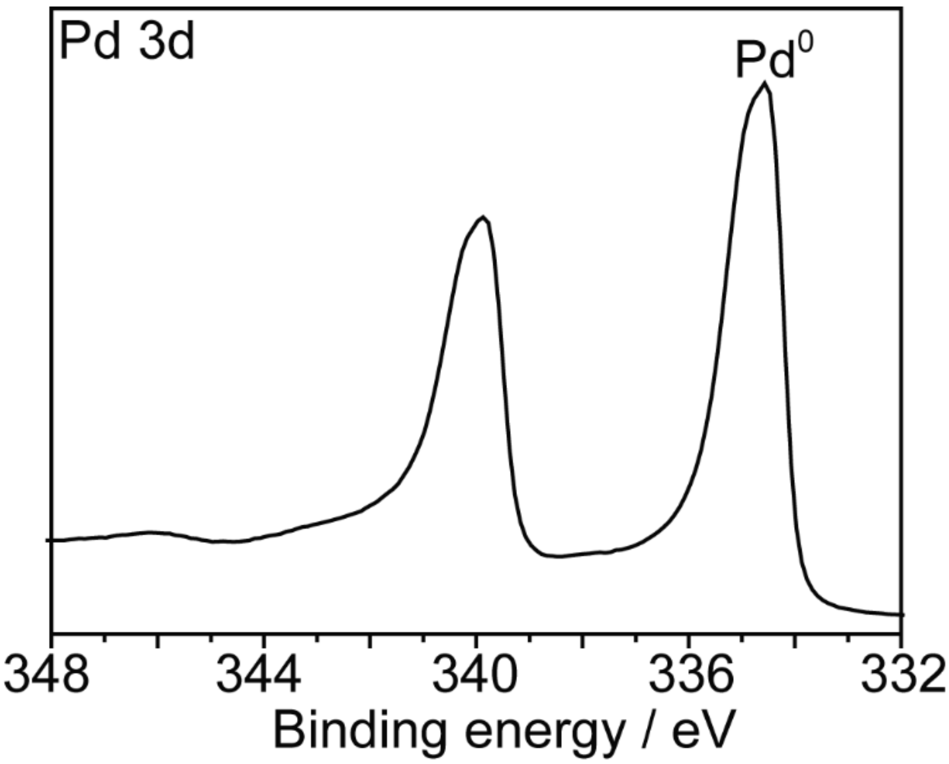

Supplementary Figure 23 HR-XPS of Pd film deposited on silicon wafer using similar parameters as those employed for Pd nanoclusters deposition in ILs. Pd *3d*$_{5/2}$ appears at 334.5 eV, which is associated to metallic Pd.[1]

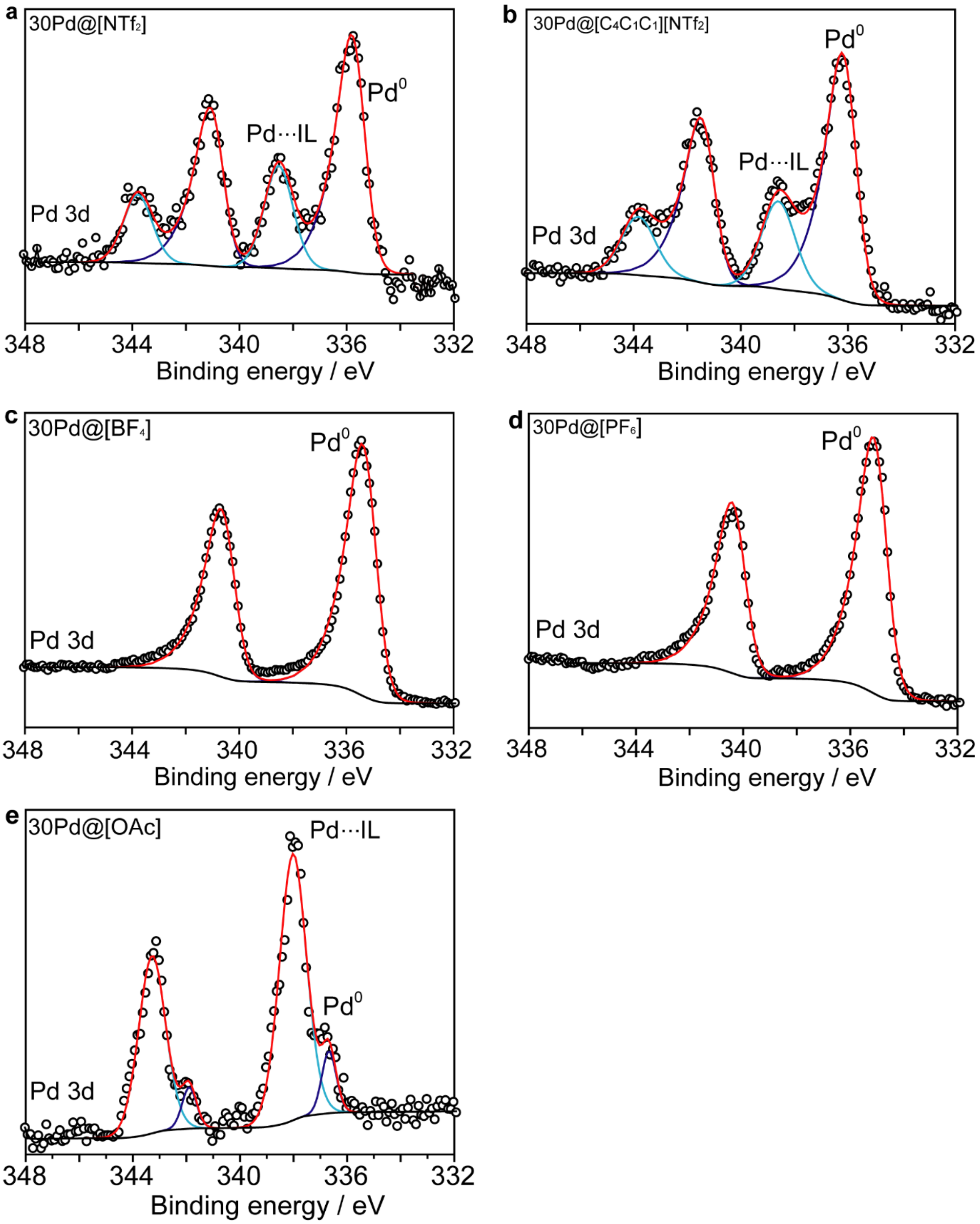

Supplementary Figure 24 HR-XPS measurements of Pd deposited for 30 min in different ionic liquids. **a** $[C_4C_1Im][NTf_2]$, **b** $[C_4C_1C_1Im][NTf_2]$, **c** $[C_4C_1Im][BF_4]$, **d** $[C_4C_1Im][PF_6]$ and **e** $[C_4C_1Im][OAc]$.

XPS analysis for Pd nanoclusters deposited in $[C_4C_1Im][PF_6]$ and $[C_4C_1Im][BF_4]$ displayed only signal associated to Pd^0. These results are opposite to our results for Pd@$[NTf_2]$. On the other hand, 30Pd@[OAc] and 30Pd@$[C_4C_1C_1Im][NTf_2]$ displayed similar behavior than 30Pd@$[NTf_2]$ displaying a peak associated to Pd^0 (*ca.* 336 eV) and another extra peak at higher binding energy (*ca.* 338 eV). Interestingly, 30Pd@[OAc] shows a much higher population of Pd species at higher binding energy

than 30Pd@[NTf_2], which suggests a stronger influence of the [OAc] anion on the Pd nanoclusters electronic environment compared to the [NTf_2] anion. The presence of an extra Pd peak at higher binding energy for 30Pd@[$C_4C_1C_1Im$][NTf_2] discards carbene formation for Pd nanoclusters, and thus reinforce the interaction between Pd species and N and SO_2 groups from the [NTf_2] anion.

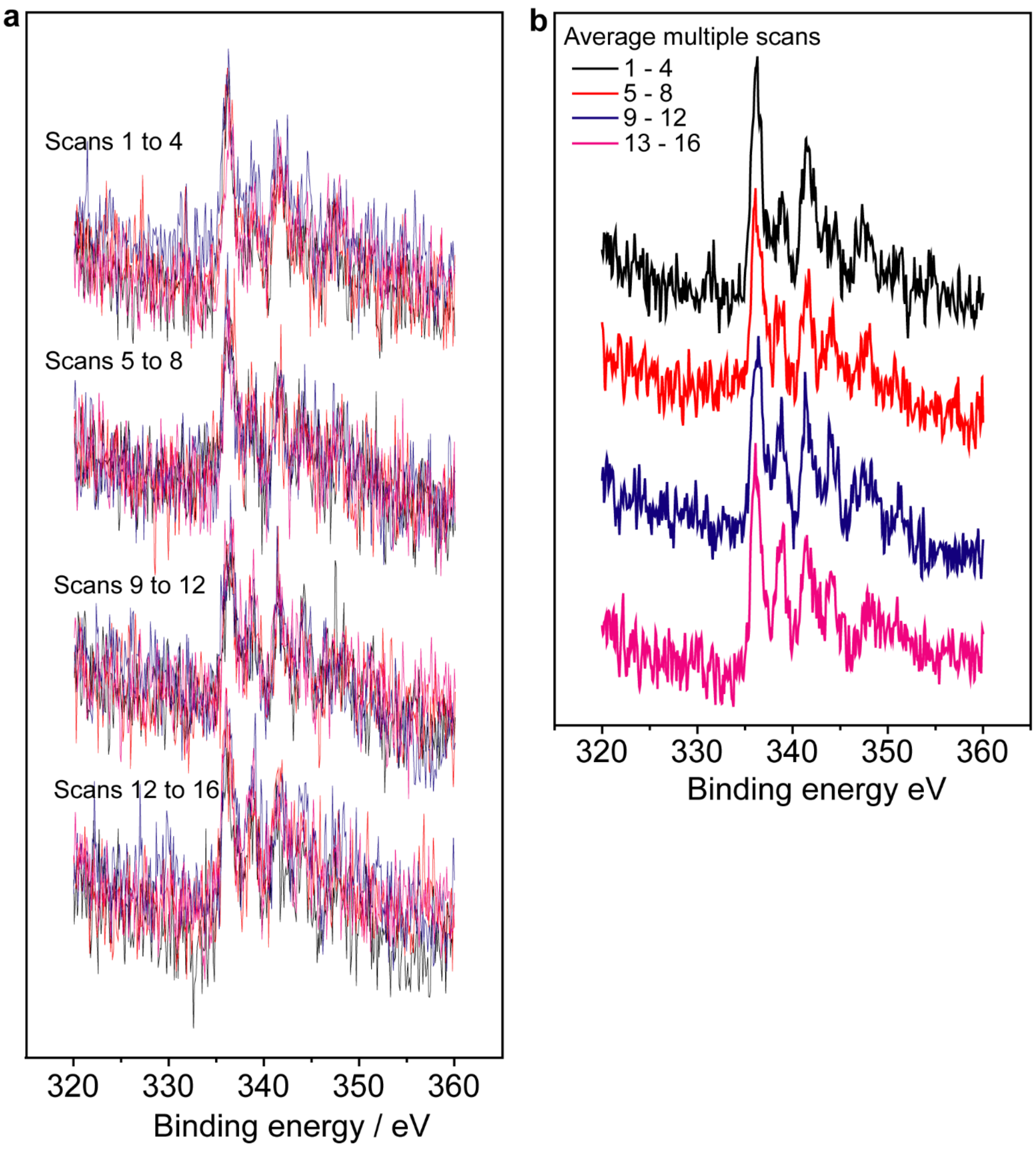

Supplementary Figure 25 Multiple XPS scans were taken at same position of the sample to evaluate possible IL surface charge for 30Pd@[NTf_2]. a The single scans were overlapped in group of 4 scans. **b** Each group of 4 scans was averaged in a single spectrum for sake of clarity. No significant changes were observed in the spectra above that suggests no charge accumulation on IL surface during the measurements. No charge correction was done in the spectra above.

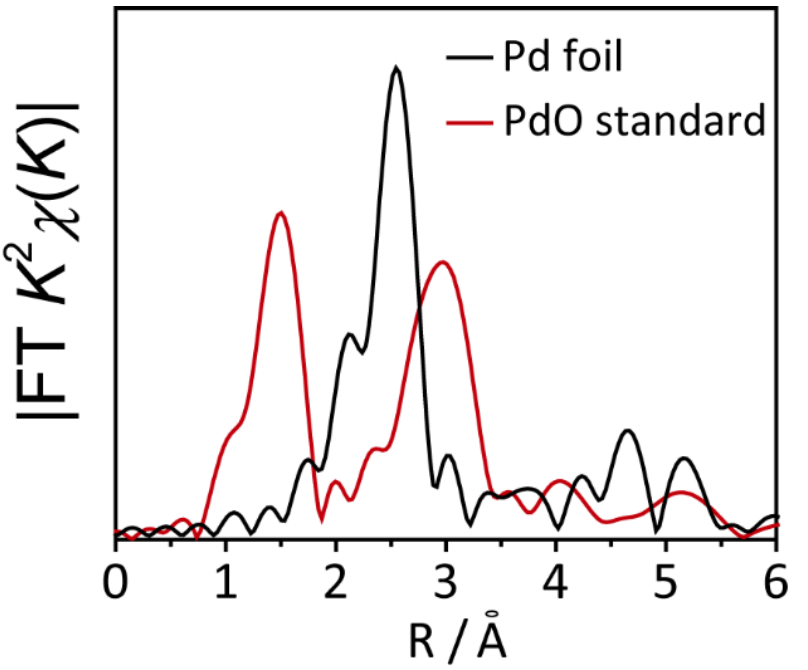

Supplementary Figure 26 EXAFS measurements of PdO and bulk Pd foil. These are standards used to compare with the results obtained for Pd@[NTf$_2$] samples.

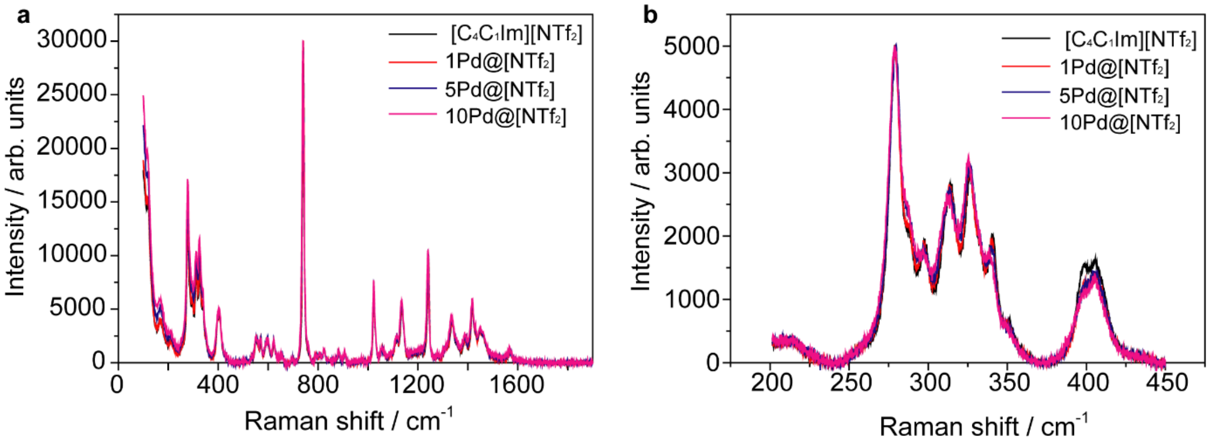

Supplementary Figure 27 Raman spectra of pure [C$_4$C$_1$Im][NTf$_2$] and with Pd deposited for 1, 5 and 10 min. a wide scan spectra and **b** high resolution spectra.

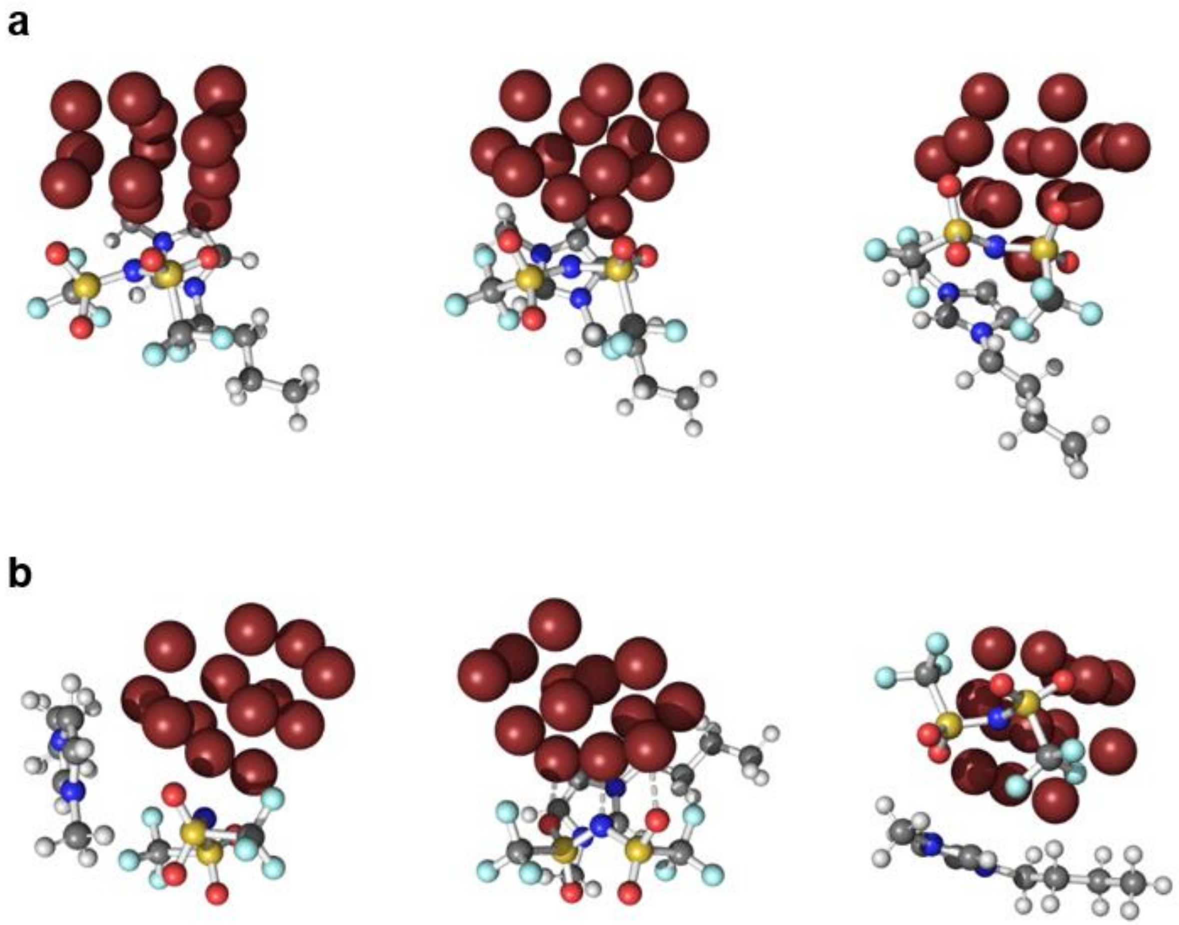

Supplementary Figure 28 DFT calculations of $[C_4C_1Im][NTf_2]$ arrangement on Pd_{13} clusters. a *cis*-conformer and **b** *trans*- conformer.

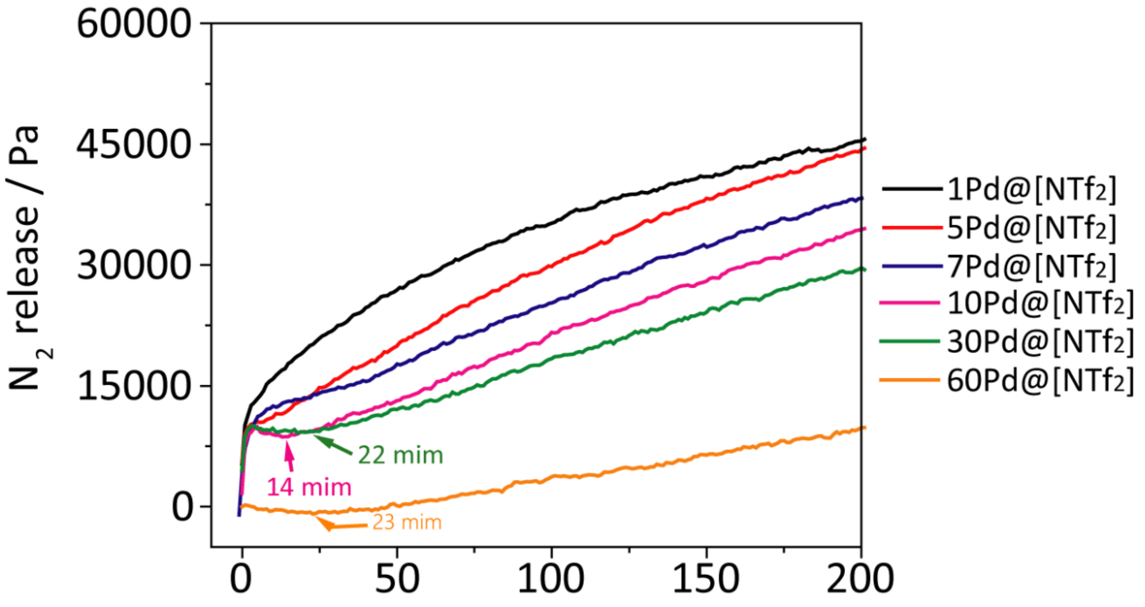

Supplementary Figure 29 N_2 evolution with time in catalytic experiments at different Pd concentrations in $[C_4C_1Im][NTf_2]$. Arrows highlight the respective incubation times for 10Pd@$[NTf_2]$, 30Pd@$[NTf_2]$ and 60Pd@$[NTf_2]$.

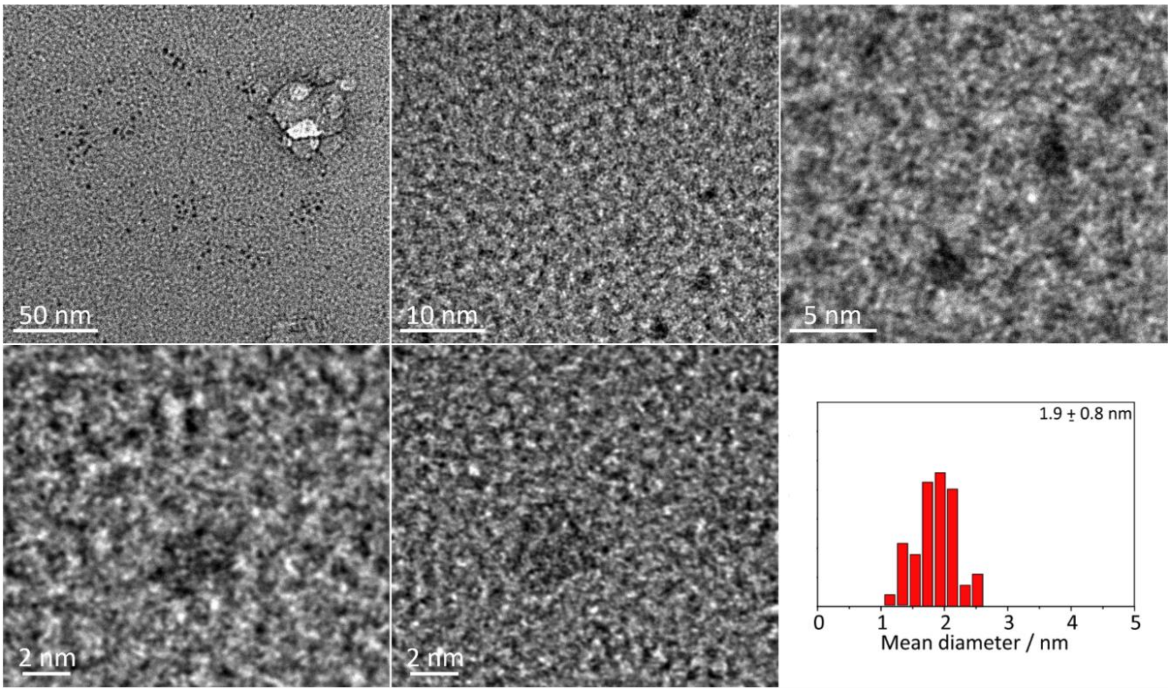

Supplementary Figure 30 TEM images and particle size distribution histogram of 5Pd@[NTf_2] after catalytic reaction. TEM measurements were performed where dynamic Pd nanoclusters were retrieved from the reaction mixture directly onto a TEM grid. Our imaging demonstrated that the dynamic clusters remain the same in size and structure within the error of the measurements.

Supplementary Table 1 Summary of magnetron sputtering parameters for the deposition of Pd into different ILs.

IL	Deposition time / min	Potential / V	Current / mA	Pd content in IL[(1)] / wt%
[C_4C_1Im][NTf_2]	1	348	287	0.03
	5	349	286	0.12
	7	349	286	0.19
	10	348	288	0.33
	20	350	285	0.66
	30	348	287	1.01
	60	349	287	2.12
[C_4C_1Im][PF_6]	5	349	287	0.12
	30	349	287	1.01
[C_4C_1Im][BF_4]	30	349	287	1.01
[$C_4C_1C_1$Im][NTf_2]	30	349	287	1.03

[(1)]Determined by ICP-OES analysis. The resulting materials were named as XPd@Y where X is Pd deposition time in min and Y is the nature of the anion for [C_4C_1Im].Y as the IL cation was kept constant. For instance, Pd species deposited for 5 min (and Pd concentration of 0.12 wt%) in [C_4C_1Im][NTf_2] would be denoted as 5Pd@[NTf_2].

Supplementary Table 2 XPS fittings for *Pd 3d* signal.

Samples	Deposition time / min	Pd $3d_{5/2}$		
		Pd^0/ eV	Pd···IL / eV	At% - Pd^0/Pd···IL
Pd film on Si*	60	334.5	-	-
$[C_4C_1Im][NTf_2]$	10	335.9	338.5	1.4
	20	335.9	338.5	1.7
	30	335.9	338.5	1.6
	60	335.9	338.5	1.9
$[C_4C_1Im][PF_6]$	30	335.0	-	-
$[C_4C_1Im][BF_4]$	30	335.2	-	-
$[C_4C_1Im][OAc]$	30	336.6	338.1	0.1
$[C_4C_1C_1Im][NTf_2]$	30	335.9	338.5	1.7

* Pd film was deposited on silicon wafer by magnetron sputtering system using a DC power of 100 W over 30 min.

Supplementary Table 3 Raman shifts of the $[NTf_2]$ anion observed in the spectra of the appraised ILs and their proposed assignment based on literature reports: *ν* (stretch), *δ* (bend), *ω* (wagging), *τ* (twisting), *ρ* (rocking), *t* (torsion), *s* (symmetric), as (antisymmetric), *ip* (in-plane), *op* (out-of-plane). All other bands observed are associated with the $[C_4C_1Im]$ cation.[2]

Raman shift / cm^{-1}		Proposed assignment
IL	Pd@$[NTf_2]$	
278.5	278.4	$\rho(CF_3)$
296.8		Unassigned
313.2	311.6	$\rho(SO_2)$
326.1	324.9	$\rho(SO_2)$
340.0		$\tau(SO_2)$
351.1		$\tau(SO_2)$
398.8		$\omega(SO_2)$
405.4	404.5	$\omega(SO_2)$
551.6	552.8	$\delta_s(SO_2)$
571.3	568.3	$\delta_{as}(CF_3)$
592.9		$\delta_{as,ip}(SO_2)$
741.2	739.1	$\nu_s(SNS)$
762.7 sh	760.8 sh	$\nu_s(SNS)$
1136.4	1133.8	$\nu_{s,ip}(SO_2)$
1242.1	1238.3	$\nu_s(CF_3)$
1337.6	1333.5	$\nu_{a,op}(CF_3)$

Supplementary Table 4 Cyclopropanation of styrene catalysed by Pd nanoclusters with different deposition times in [C_4C_1Im][NTf_2].[a,b]

Pd:IL (%w/w)	Conversion (%)	Selectivity (%)	Yield (%)	TON
0.02	79	93	69	138
0.12	89	92	76	152
0.19	83	93	73	146
0.33	67	94	60	120
0.66	63	93	55	110
1.01	58	94	51	102
2.12	46	93	40	80

[a] Reagents and conditions: catalyst (0.5 mol% Pd), EDA (1 mmol), styrene (5 mmol), R.T., 24 h, CH_2Cl_2 (10 mL), one pot. [b] Conversion and yield of cyclopropane product determined by ^{1}H NMR spectroscopy by the use of 1,2-dibromoethane as an internal standard.

Supplementary Table 5 Summary of styrene cyclopropanation by various catalytic Pd@IL systems.

Entry	Catalyst	Conversion (%)[a]	Selectivity (%)	Yield (%)[a]
1	5Pd@[NTf_2]	89	92	76
2	5Pd@[PF_6]	55	86	45
4	30Pd@[NTf_2]	58	94	51
5	30Pd@[PF_6]	41	92	35

Reagents and conditions: catalyst (0.5 mol% Pd), EDA (1 mmol), styrene (5 mmol), CH_2Cl_2 (10 mL), R.T., 24 h, one pot.[a] Conversion and yield of cyclopropane product determined by ^{1}H NMR spectroscopy by the use of 1,2-dibromoethane as internal standard.

Supplementary Table 6 Summary of the Hg(0) test using various catalytic Pd@IL systems.

Entry	Catalyst	Conversion (%)[a]	Selectivity (%)	Yield (%)[a]
1	1Pd@[NTf_2]	77	90	69
2	5Pd@[NTf_2]	51	89	41
3	7Pd@[NTf_2]	<5%	n.a.	n.a.
4	10Pd@[NTf_2]	0	-	-
5	20Pd@[NTf_2]	0	-	-
6	30Pd@[NTf_2]	0	-	-
7	60Pd@[NTf_2]	0	-	-
8	5Pd@[PF_6]	0	-	-

Reagents and conditions: catalyst (0.5 mol% Pd), EDA (1 mmol), styrene (5 mmol), CH_2Cl_2 (10 mL), R.T., 24 h, one pot.[a] Conversion and yield of cyclopropane product determined by ^{1}H NMR spectroscopy by the use of 1,2-dibromoethane as internal standard. [b] Deposition time: 5 min.[c] Deposition time: 30 min.

Supplementary Table 7 Summary of DCT tests undertaken with 5Pd@NTf_2 and 30Pd@NTf_2.

Entry	Catalyst	DCT (eq)	Conversion (%)[a]	Selectivity (%)
1	5Pd@[NTf_2]	0	89	92
2	5Pd@[NTf_2]	1	70	>90
3	5Pd@[NTf_2]	2	67	>90
4	30Pd@[NTf_2]	0	58	>90
5	30Pd@[NTf_2]	1	69	>90
6	30Pd@[NTf_2]	2	55	>90

Reagents and conditions: catalyst (0.5 mol% Pd), EDA (1 mmol), styrene (5 mmol), CH_2Cl_2 (5 mL), DCT (0.5 mol%, 1 eq, respectively 1 mol%, 2 eq), R.T., 24 h, one pot.[a] Conversion and yield of cyclopropane product determined by ^{1}H NMR spectroscopy by the use of 1,2-dibromoethane as internal standard.

Supplementary Table 8 Summary of Filtration tests undertaken with 5Pd@NTf_2 and 30Pd@NTf_2.

Entry	Catalyst	Conversion (%)[a]	Selectivity (%)	Yield (%)[a]
1	5Pd@[NTf_2]	0	-	-
2	30Pd@[NTf_2]	0	-	-

Reagents and conditions: catalyst (0.5 mol% Pd), EDA (1 mmol), styrene (5 mmol), CH_2Cl_2 (5 mL), R.T., 24 h, one pot.[a] Conversion and yield of cyclopropane product determined by ^{1}H NMR spectroscopy by the use of 1,2-dibromoethane as internal standard.

Supplementary Table 9 Summary of recycling tests undertaken with 5Pd@NTf$_2$ and 30Pd@NTf$_2$.

Entry	catalyst	Cycle	Selectivity (%)	Conversion[a] (%)	Pd loading (mol%)	Leaching (%)	TON
1	5Pd@[NTf$_2$]	1	90	>95	0.5	4	200
2	5Pd@[NTf$_2$]	2	>95	>95	0.48	4	210
3	5Pd@[NTf$_2$]	3	62	83	0.46	4	180
4	30Pd@[NTf$_2$]	1	80	60	0.5	2	120
5	30Pd@[NTf$_2$]	2	88	55	0.49	2	112
6	30Pd@[NTf$_2$]	3	85	63	0.48	2	131

Reagents and conditions: catalyst (0.5 mol% Pd), EDA (1 mmol), styrene (5 mmol), CH_2Cl_2 (5 mL), DCT (0.5 mol%, 1 eq, respectively 1 mol%, 2 eq), R.T., 24 h, one pot.[a] Conversion and yield of cyclopropane product determined by ^{1}H NMR spectroscopy by the use of 1,2-dibromoethane as internal standard.

Supplementary Notes 1

Carbene transfer reactions from ethyl diazoacetate to C=C bonds catalysed by Pd@IL system

Pd nanoclusters deposited in ILs were used as catalyst without any pre-treatment in cyclopropanation reactions by carbene transfer from the commercially available ethyl diazoacetate (EDA) to alkenes (Supplementary Figure 31).

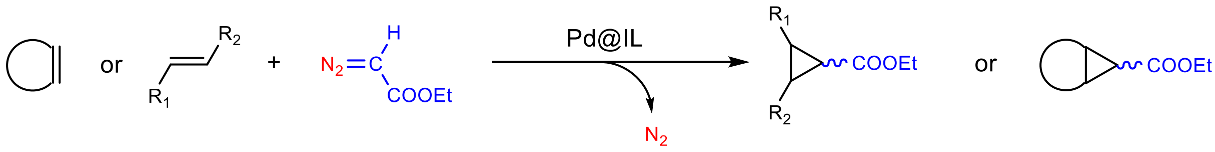

Supplementary Figure 31 Formation of cyclopropanes by carbene transfer from EDA to alkenes catalysed by Pd@[NTf$_2$] systems.

In order to optimise the reaction conditions, we studied the catalytic activity of Pd nanoclusters deposited for 30 min in [C$_4$C$_1$Im][NTf$_2$] in a set of screening experiments for the cyclopropanation reaction of styrene with EDA as carbene source (Supplementary Table 10). The analysis of the reaction mixture revealed the formation of *cis*- and *trans*-cyclopropane products and the presence of two side products, diethyl fumarate and maleate, which were generated in a competitive process also mediated by the catalyst (Supplementary Figure 32).

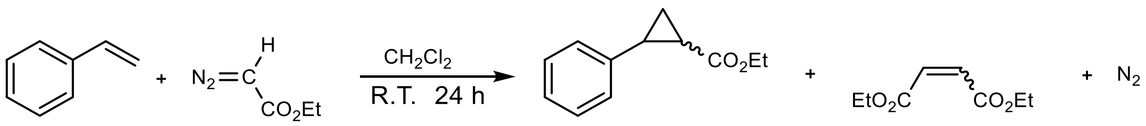

Supplementary Figure 32 Cyclopropanation of styrene catalysed by Pd nanoclusters deposited for 30 min in [C$_4$C$_1$Im][NTf$_2$].

An equimolar mixture of styrene and EDA in dichloromethane with 0.5 mol% catalyst loading provided 84% selectivity towards the cyclopropane product (mixture of *cis*- and *trans*-) at R.T. in 24 h, albeit

with low conversion (entry 1). Increasing the amount of styrene to 5 eq., 58% conversion with 94% selectivity was observed (entry 2). However, an increase in the catalyst loading to 1 mol% did not improve the results (entry 3). Interestingly, a decrease in the amount of solvent led to an increase in the conversion (entry 4), but the observed selectivity was slightly lower (90%). Furthermore, the slow addition of EDA during 24 h did not increase the selectivity (entry 5). It is worth noting that the use of non-purified solvent and substrates did not involve a decrease in the activity, highlighting the robustness of the catalytic system. With these optimised reaction parameters (styrene (5 eq.), catalyst (0.5 mol% Pd), 24 h, R.T.), 79% conversion and 90% selectivity were achieved with 5 mL of CH_2Cl_2 (entry 4), while in terms of selectivity 58% conversion and 94% selectivity were accomplished for 10 mL of CH_2Cl_2 (entry 2).

Supplementary Table 10 Optimisation Parameters for the cyclopropanation of styrene catalysed by Pd nanoclusters deposited for 30 min in [C_4C_1Im][NTf_2].[a]

Entry	[cat] (mol% Pd)	[styrene]	Conversion (%)[b]	Selectivity (%)	Yield (%)[b]
1	0.5	1 eq.	32	84	23
2	0.5	5 eq.	58	94	51
3	1	5 eq.	60	91	51
4[c]	0.5	5 eq.	79	90	65
5[d]	0.5	5 eq.	76	91	64
6[e]	0.5	5 eq.	70	92	60

[a] Reagents and conditions: EDA (1 mmol), CH_2Cl_2 (10 mL), R.T., 24 h, one pot. [b] Conversion and yield of cyclopropane product determined by ^1H NMR spectroscopy by the use of 1,2-dibromoethane as internal standard. [c] Using CH_2Cl_2 (5 mL). [d] Slow addition. [e] Using CH_2Cl_2 (5 mL) and reagents without any purification.

A series of additional control experiments was carried out in order to determine the importance of Pd@[NTf_2] system to the observed catalytic activity (Supplementary Table 11). First, very low activity was observed by the use of palladium supported on carbon (Pd/C, entry 1), while the IL [C_4C_1Im][NTf_2] was totally inactive (entry 2). In addition, a mixture of Pd/C and [C_4C_1Im][NTf_2] showed very low activity (entry 3). Next, we found that a mixture of palladium acetate ($Pd(OAc)_2$) and IL ([C_4C_1Im][NTf_2]) was less active than Pd nanoclusters deposited for 30 min in [C_4C_1Im][NTf_2] under the optimised reaction conditions (entries 4 and 5, respectively).

Supplementary Table 11 Summary of the attempted cyclopropanation of styrene using various catalytic systems

Entry	Catalyst	Conversion (%)[d]	Selectivity (%)	Yield (%)[d]
1[a]	Pd/C	9	96	8
2[b]	$[C_4C_1Im][NTf_2]$	0	--	0
3[c]	Pd/C + $[C_4C_1Im][NTf_2]$	10	90	9
4	$Pd(OAc)_2$ + $[C_4C_1Im][NTf_2]$	37	92	34
5[d]	$30Pd@[NTf_2]$	58	94	51

Reagents and conditions: EDA (1 mmol), styrene (5 mmol), CH_2Cl_2 (10 mL), R.T., 24 h, one pot.[a] Pd/C (0.5 mol% Pd).[b] 50 mg $[C_4C_1Im][NTf2]$.[c] Pd/C (0.5 mol% Pd) + 50 mg $[C_4C_1Im][NTf_2]$.[d] Conversion and yield of cyclopropane product determined by ^1H NMR spectroscopy by the use of 1,2-dibromoethane as internal standard.

Following optimisation of the reaction conditions, we studied the substrate scope in order to evaluate the potential of Pd nanoclusters deposited for 30 min in $[C_4C_1Im][NTf_2]$ as cyclopropanation catalyst (Supplementary Table 12). In terms of selectivity, the reaction appears to be very general and, in nearly all cases, high selectivities toward the cyclopropanation product were obtained. Nevertheless, we observed differences in the activity depending on the reactant. The nature of the substituents on the aromatic ring did not affect the efficiency of the reaction, since styrene derivatives with electron-donor groups on the phenyl ring gave similar yields of cyclopropane product as those observed for electron-withdrawing substituents (entries 1-3), although a slight increase in selectivity was observed for the former (entry 1). The results also indicate a steric influence of the substituents in the substrate that hinders the interaction of the catalyst with the alkene functionality and leads to a lower conversion and selectivity (entry 4). In addition, the catalytic system is highly tolerant to aliphatic alkenes, but the conversions and selectivities observed are lower than those found for styrene derivatives (entries 4-7). Finally, of particular interest is the cyclopropanation of cyclooctene (entry 6), since this transformation can be considered challenging due to the steric impediment shown by the substrate. Interestingly, the catalytic reaction was carried out with reasonable conversion and selectivity (73 and 89 %, respectively).

Supplementary Table 12 Cyclopropanation of different olefins catalysed by Pd nanoclusters deposited for 30 min in [C_4C_1Im][NTf_2].[a]

Entry	Substrate	Conversion (%)[b]	Selectivity (%)	Yield (%)[b]
1	*p*-methoxystyrene	85	96	78
2	styrene	79	90	65
3	*p*-chlorostyrene	88	91	73
4	α-methylstyrene	17	43	5
5	1-hexene	53	89	44
6	cyclooctene	73	89	58
7	n-butyl vinyl ether	25	83	18

[a] Reagents and conditions: catalyst (0.5 mol% Pd), EDA (1 mmol), olefin (5 mmol), CH_2Cl_2 (5 mL), R.T., 24 h, one pot.[b] Conversion and yield of cyclopropane product determined by ^{1}H NMR spectroscopy by the use of 1,2-dibromoethane as internal standard.

Supplementary Methods

Synthesis and characterization of 1-Butyl-3-methylimidazolium bis(trifluoromethylsulfonyl)imide, [C_4C_1Im][NTf_2][3,4]

To a solution of 1-butyl-3-methylimidazolium chloride[4-6] (2.08 g, 11.9 mmol) in water (10 mL), a solution of lithium bis(trifluoromethane)sulfonimide (4.11 g, 14.3 mmol) in water (10 mL) was added dropwise and the mixture stirred at 0 °C. The reaction mixture was gradually allowed to warm up to room temperature (R.T.) and left stirring for 24 h. Dichloromethane (30 mL) was added to recover the IL and washed with cold water (5 x 10 mL). The organic layer was collected, and the solvent removed *in vacuo* (1 mbar). The product was dried *in vacuo* ($p \leq 10^{-3}$ mbar) overnight at 50°C to yield a colorless liquid (9.82 g, 98.2 %).

^{1}H NMR (400 MHz, DMSO-d6): δ 9.10 (br. s, 1 H), 7.75 (t, J = 1.8 Hz, 1 H), 7.68 (t, J = 1.8 Hz, 1 H), 4.16 (t, J = 7.4 Hz, 2 H), 3.85 (s, 3 H), 1.77 (quint, J = 7.4 Hz, 2 H), 1.26 (sxt, J = 7.4 Hz, 2 H), 0.90 (t, J = 7.4 Hz, 3 H); ^{13}C NMR (100 MHz, DMSO-d6): δ 136.5, 123.6, 122.2, 119.9 (q, $^1J_{C-F}$ = 322 Hz, 1 C), 48.5, 35.7, 31.3, 18.7, 13.2; ^{19}F NMR (376 MHz, DMSO-d6): δ -78.8 (s, 6 F). ESI-MS (+ve) $[C_8H_{15}N_2]^+$: calcd 139.1230 m/z found 139.1243 m/z. ESI-MS (-ve) $[C_2F_6NO_4S_2]^-$: calcd 279.9173 m/z, found 279.9190 m/z.

Synthesis and characterisation of 1-Butyl-3-methylimidazolium hexafluorophosphate, $[C_4C_1Im][PF_6]$[4]

To a solution of 1-butyl-3-methylimidazolium chloride[4-6] (3.07 g, 17.6 mmol) in water (10 mL), a solution of potassium hexafluorophosphate (3.89 g, 21.1 mmol) in water (10 mL) was added dropwise and the mixture stirred at 0 °C. The reaction mixture was gradually allowed to warm up to R.T. and left stirring for 24 h. Dichloromethane (30 mL) was added to recover the IL and washed with cold water (5 x 10 mL). The organic layer was collected, and the solvent removed *in vacuo* (1 mbar). The product was dried *in vacuo* ($p \leq 10^{-3}$ mbar) overnight at 50 °C to yield a colorless liquid (4.23 g, 84.6 %).

^{1}H NMR (400 MHz, DMSO-d_6): δ 9.08 (br. s, 1 H), 7.74 (t, J = 1.8 Hz, 1 H), 7.68 (t, J = 1.8 Hz, 1 H), 4.15 (t, J = 7.4 Hz, 2 H), 3.84 (s, 3 H), 1.77 (quint, J = 7.4 Hz, 2 H), 1.26 (sxt, J = 7.4 Hz, 2 H), 0.90 (t, J = 7.4 Hz, 3 H); ^{13}C NMR (100 MHz, DMSO-d_6): δ 136.5, 123.6, 122.3, 48.5, 35.7, 31.3, 18.8, 13.2; ^{31}P NMR (160 MHz, DMSO-d_6): δ -144.2 (spt, $^{1}J_{P\text{-}F}$ = 712 Hz, 1 P); ^{19}F NMR (376 MHz, DMSO-d_6): δ -70.2 (d, $^{1}J_{F\text{-}P}$ = 712 Hz, 6 F). ESI-MS (+ve) $[C_8H_{15}N_2]^+$: calcd 139.1230 m/z found 139.1199 m/z.

Synthesis and characterisation of 1-Butyl-3-methylimidazolium tetrafluoroborate, $[C_4C_1Im][BF_4]$[4]

To a solution of 1-butyl-3-methylimidazolium chloride[4-6] (3.86 g, 22.1 mmol) in water (10 mL), a solution of sodium tetrafluoroborate (2.91 g, 26.5 mmol) in water (10 mL) was added dropwise and the mixture stirred at 0 °C. The reaction mixture was gradually allowed to warm up to R.T. and left stirring for 24 h. Dichloromethane (30 mL) was added to recover the IL and washed with cold water (5 x 10 mL). The organic layer was collected, and the solvent removed *in vacuo* (1 mbar). The product was dried *in vacuo* ($p \leq 10^{-3}$ mbar) overnight at 50 °C to yield a colorless liquid (3.62 g, 72.3%).

^{1}H NMR (400 MHz, $CDCl_3$): δ 8.83 (br. s, 1 H), 7.33 (t, J = 1.8 Hz, 1 H), 7.28 (t, J = 1.8 Hz, 1 H), 4.18 (t, J = 7.4 Hz, 2 H), 3.96 (s, 3 H), 1.86 (quint, J = 7.4 Hz, 2 H), 1.37 (sxt, J = 7.4 Hz, 2 H), 0.95 (t, J = 7.4 Hz, 3 H); ^{13}C NMR (100 MHz, $CDCl_3$): δ 136.8, 123.7, 122.1, 50.0, 36.5, 32.0, 19.5, 13.5; ^{19}F NMR (376 MHz, $CDCl_3$): δ -151.6 (q, J = 1.1 Hz), -151.5 (br. s). ESI-MS (+ve) $[C_8H_{15}N_2]^+$: calcd 139.1230 m/z, found 139.1212 m/z.

Synthesis and characterization of 1,2-Dimethyl-3-butyl-imidazolium bis(trifluoromethylsulfonyl)imide, $[C_4C_1C_1Im][NTf_2]$[4]

To a solution of 1,2-dimethyl-3-butyl-imidazolium chloride[4-6] (2.18 g, 11.5 mmol) in water (10 mL), a solution of lithium bis(trifluoromethane)sulfonimide (3.98 g, 13.8 mmol) in water (10 mL) was added dropwise and the mixture stirred at 0 °C. The reaction mixture was gradually allowed to warm up to R.T. and left stirring for 24 h. Dichloromethane (30 mL) was added to recover the IL and washed with cold water (5 x 10 mL). The organic layer was collected, and the solvent removed *in vacuo* (1 mbar).

The product was dried *in vacuo* ($p \leq 10^{-3}$ mbar) overnight at 50 °C to yield a colorless liquid (4.89 g, 97.7%).

^{1}H NMR (400 MHz, DMSO-d_6): δ 7.64 (d, J = 2.0 Hz, 1 H), 7.61 (d, J = 2.0 Hz, 1 H), 4.10 (t, J = 7.4 Hz, 2 H), 3.74 (s, 3 H), 2.57 (s, 3 H), 1.69 (quin, J = 7.4 Hz, 2 H), 1.28 (sxt, J = 7.4 Hz, 2 H), 0.91 (t, J = 7.4 Hz, 3 H); ^{13}C NMR (100 MHz, DMSO-d_6): δ 144.2, 122.3, 120.9, 119.9 (q, $^{1}J_{C\text{-}F}$ = 322 Hz, 1 C), 47.3, 34.6, 31.2, 18.9, 13.4, 9.1; ^{19}F NMR (376 MHz, DMSO-d_6): δ -78.8 (s, 6 F). ESI-MS (+ve) $[C_9H_{17}N_2]^+$: calcd 153.1386 m/z, found 153.1392 m/z. ESI-MS (-ve) $[C_2F_6NO_4S_2]^-$: calcd 279.9173 m/z, found 279.9181 m/z.

Synthesis of 5,6,11,12-tetrahydrodibenzo[*a,e*]cyclooctene

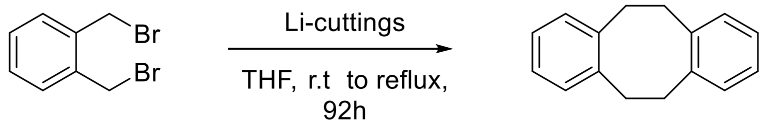

In a glove box: in a Schlenck flask equipped with a stirrer bar, *α,α'*-dibromo-*o*-xylene (10.6 g, 40.2 mmol, 1 eq) are dissolved in THF (16 mL). The freshly prepared solution is transferred to a Schlenck line and added to a suspension containing 700 mg Li cuttings (101.6 mmol, 2.5 eq) using a transfer cannula. The rate of transfer was controlled using pressure exchanges according to standard Schlenck line techniques and lasted 30 min. Afterwards, the resulting reaction mixture is stirred under reflux for 2 h. Afterwards, the reaction is cooled to R.T. and additionally stirred for 18 h. The reaction progress was checked *via* TLC. After 18 h, a new batch of 700 mg Li cutting (101.6 mmol, 2.5 eq) is added. Then, the reaction is stirred for 72 h at 60°C. The resulting red/brown solution is cooled to R.T. and filtered through a celite pad. The pad is washed with THF (200 mL) and the remaining Li cuttings are neutralised with isopropanol. The filtrate is dispersed in CH_2Cl_2 (400 mL) and filtered through a small pad of Al_2O_3, which is washed with CH_2Cl_2 (400 mL). The collected filtrate is reduced under vacuum. The crude product is purified *via* flash chromatography (conditions: 80 g SiO_2, 3 cm diameter, 50 cm height, R_f = 0.25) using petroleum ether 40-60 to yield 5,6,11,12-Tetrahydrodibenzo[a,e]cyclooctene as colorless plates. Note: No impurities are observed after the product. *α,α'*-Dibromo-*o*-xylene appears prior to the product (R_f = 0.31) and forms a mixed fraction with 5,6,11,12-tetrahydrodibenzo[a,e]cyclooctene.

Yield: 1.32 g, 6.34 mmol, 16%

^{1}H-NMR (400 MHz, $CDCl_3$): 7.04-6.93 (m, 8H), 3.06 (s, 8H).

^{13}C-NMR (100 MHz, $CDCl_3$): 140.6, 129.7, 126.1, 35.2.

Data in accordance with those reported previously.[7,8]

Synthesis of 5,11-dibromo-5,6,11,12-tetrahydrodibenzo[*a,e*]cyclooctene

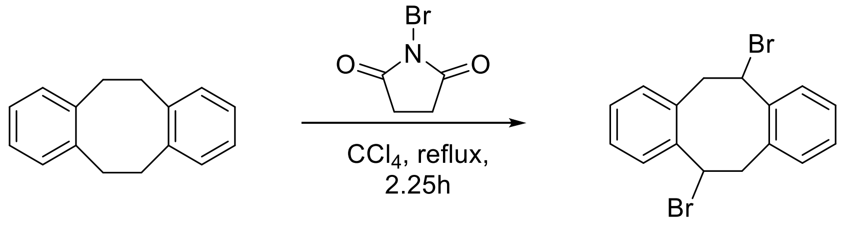

In a round bottom flask equipped with a stirrer bar and a condenser, 5,6,11,12-tetrahydrodibenzo[a,e]cyclooctene (1.32 g, 6.34 mmol, 1 eq) and *N*-bromosuccinimide (2.40 g, 13.5 mmol, 2.1 eq) are dissolved in CCl_4 (18 mL) and stirred under reflux for 2 h during 15 min. The reaction mixture is filtered whilst still hot and the solvent removed under reduced pressure. The resulting crude product is washed with 2 x 25 mL H_2O and dried under vacuum for 18h to yield 5,11-dibromo-5,6,11,12- tetrahydrodibenzo[a,e]cyclooctene as a yellow solid. The product is used in the next step without further purification.

Yield: 1.33 g, 3.63 mmol, 57%.

^{1}H-NMR (400 MHz, $CDCl_3$): 7.18-6.92 (m, 8H), 5.91 (dd, J=11.2 Hz, 9.88 Hz, 2H), 4.25 (dd, J =13.6 Hz, 2H), 3.95 (dd, J= 8.5 Hz, 8.6Hz, 2H).

Data in accordance with those reported previously.[7,8]

Synthesis of dibenzo[a,e]cyclooctene (DCT)

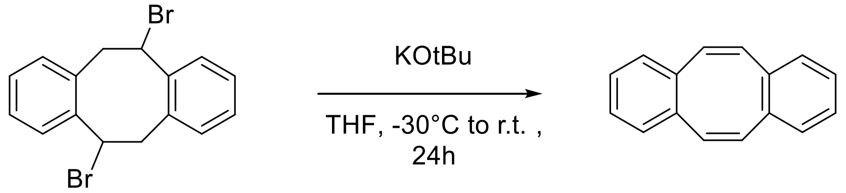

In a glove box, 5,11-dibromo-5,6,11,12-tetrahydrodibenzo[*a,e*]cyclooctene (1.33 g, 3.63 mmol, 1 eq) and potassium *tert*-butoxide (3.15 g, 28 mmol, 7.7 eq) are dissolved in different Schlenck flasks containing THF (11 mL), respectively THF (12 mL). Both solutions are transferred to a Schlenck line and the solution containing potassium *tert*-butoxide is cooled to -30°C using brine. Then, the solution containing 5,11-dibromo-5,6,11,12- tetrahydrodibenzo[a,e]cyclooctene is transferred to the solution containing potassium *tert*-butoxide over a period of 40 min using a transfer cannula. The flow is controlled using standard Schlenck line techniques. The resulting reaction mixture quickly turned brown, and the mixture was warmed to R.T. and left under stirring for 18 h. After 18 h, the reaction progress is checked using TLC and H_2O (1.1 mL) is added. The reaction mixture is filtered through a celite pad and the celite pad washed with Et_2O (400 mL). The resulting red solution is dried over $MgSO_4$, filtered and the solvent is removed under reduced pressure. The dark red/brown product is purified using flash chromatography (conditions: 80 g SiO_2, 3 cm diameter, 50 cm height, R_f = 0.30).

The resulting colorless solid is recrystallised in cold pentane yielding dibenzo[*a,e]*cyclooctene (**DCT**) as colorless needles.

Yield: 140 mg, 0.69 mmol, 19%.

^{1}**H-NMR** (400 MHz, $CDCl_3$): 7.18 (m, 4H), 7.09 (m, 4H), 6.78 (s, 4H).

^{13}**C-NMR** (100 MHz, $CDCl_3$): 137.1, 133.3, 129.1, 126.8.

Data in accordance with those reported previously.[7,8]

Optimisation of potential applied in magnetron sputtering Pd depositions

The classical molecular dynamics simulations were conducted with the PARCAS code[9]. The simulation setup included 4000 atoms in an FCC lattice. The simulation cell was prepared by first applying an isothermal-isobaric ensemble at 10 K and 100 Pa for 100 ps with periodic boundaries in all directions, allowing the structure to relax. This was followed by 100 ps in a canonical ensemble at 10 K fixing the bottom layer of the cell and applying periodic boundaries in x- and y-directions with an open 111 surface facing up. After this the irradiation events were simulated with the Berendsen thermostat[10] applied at the periodic edges, including a few atomic rows, to mimic energy dissipation in an infinite cell and avoid excess heat passing through the boundaries.

The Ar atom was placed 10 Å above the 111-surface of the Pd-cube, and given an initial energy of 280, 320, 400, 640 and 800 eV in a small angle of 5 degrees off the normal of the surface. The small angle was included to take into account the ions hitting the target in slightly non-perpendicular angles in the experiments. The azimuthal angle was randomised to mimic experimental conditions. For each energy, 500 impacts were simulated with a randomised impact point. Each recoil was shot towards a pristine target with the simulation time set to 5000 fs, allowing the system to reach a local minimum after the impact before the final configurations were analysed.

The interactions between the Pd atoms were modelled using the embedded atom method (EAM) potential for the interactions between Pd atoms and smoothly joined to the universal ZBL repulsive potential[11] at close atomic separations. The interactions between the Ar and Pd atoms were modelled with the ZBL potential. The inelastic energy losses were included in the model through electronic stopping for all atoms, excluding the sputtered atoms, when the kinetic energy of the atom exceeded a threshold of 5 eV. The magnitude of the stopping was given by SRIM (http://www.srim.org).

Synthesis Pd nanoclusters in ionic liquid via magnetron sputtering

Pd species were deposited into ILs using a bespoke AJA magnetron sputtering system with a load-lock sample transfer facility coupled to a glove-box. The Pd target (99.995 %) was purchased from *AJA*

International. IL samples were loaded through a glove-box into the magnetron sputtering system to avoid the presence of moisture.

In a typical experiment, anhydrous IL (0.750 g) is placed in a petri dish whilst in the glove box. The sample was transferred to a load-lock under a N_2 environment, pumped-down to a background pressure of 5.3×10^{-5} Pa for (*ca.* 1 hr) and then transferred to the main chamber, which reached a background pressure of 5.3×10^{-6} Pa for *ca.* 1 hr. The power and working pressure used in all depositions were 100 W and 4.0×10^{-1} Pa (Argon - 99.9999 %), respectively. The other parameters used during depositions are described in Supplementary Table 1. After all depositions were carried out, the samples were kept under an inert atmosphere in the glove-box.

Characterisation of Pd species in ionic liquids

Transmission electron microscopy (TEM) measurements were performed using a JEOL 2100F FEG-TEM operated with an accelerating voltage of 200 kV. AC-STEM measurements were performed using a JEOL 2100F scanning transmission electron microscope with a CEOS aberration corrector operated with an accelerating voltage of 200 kV. AC-STEM and TEM samples were prepared in the following manners: *i*- the samples were prepared by sonicating the Pd@IL samples under ultrasound for 10 min and collecting a thin layer of the ionic liquid containing of Pd nanoclusters by using a 3 mm diameter Cu hoop dipped in the sample. This thin layer was screened on the surface of a Holey Carbon copper grid; ii- copper mesh, holey carbon film TEM grids (Agar Scientific, UK) were Ar glow discharged (Agar Turbo Coater, 0.2 mbar, 5 mA, 10 secs) before the addition of Pd@IL (10 µL). The Pd@IL suspension was kept on the grid for 2 min before the excess was removed using filter paper. This approach allowed the formation of 'pools' of IL within the holes of the holey carbon, providing an effective contrast free region to observe the palladium structures. The size distribution histograms were obtained by counting 150 particles from a minimum of 4 images for each sample; Pd clusters smaller than 1 nm were not counted.

Matrix-assisted laser desorption/ionisation time-of-flight (MALDI-TOF) mass spectrometry data were acquired using an ultrafleXtreme instrument (Bruker Daltonics, Bremen, Germany), which is equipped with a Smartbeam-II Nd:YAG laser (λ = 355 nm). The samples were either mixed neat with the matrix solution or first diluted 1:10 (v/v) with acetonitrile (CH_3CN; HPLC grade, Fisher, UK). The matrix used was trans-2-[3-(4-tert-butylphenyl)-2-methyl-2-propenylidene]malononitrile (DCTB; Insight Biotechnology, UK) made up in HPLC CH_3CN at 20mg/mL; sample and matrix were mixed in either ratios of 1:5 or 1:24 (v/v). Data were acquired in either positive-linear or -reflectron modes using FlexControl software v3.4, while post-acquisition processing of data was performed by FlexAnalysis software v3.4. All the measurements were performed at the National Mass Spectrometry Facility in Swansea-UK.

X-ray photoelectron spectroscopy (XPS) measurements were performed using a Kratos AXIS Ultra DLD instrument. The chamber pressure during the measurements was 6.7×10^{-7} Pa. Wide energy range survey scans were collected at pass energy of 80 eV in hybrid slot lens mode and a step size of 0.5 eV, for 20 min. High-resolution data on the Pd 3d, C 1s, N 1s, O 1s, S 2p and F 1s photoelectron peaks was collected at a pass energy of 20 eV over energy ranges suitable for each peak, and collection times of 5 min, step sizes of 0.1 eV. The charge neutraliser filament was used to prevent the sample charging over the irradiated area. The X-ray source was a monochromated Al Kα emission, run at 10 mA and 12 kV (120 W). The energy range for each 'pass energy' (resolution) was calibrated using the Kratos Cu $2p_{3/2}$, Ag $3d_{5/2}$ and Au $4f_{7/2}$ three-point calibration method. The transmission function was calibrated using a clean gold sample method for all lens modes and the Kratos transmission generator software within Vision II. The data were processed with CASAXPS (Version 2.3.17). The high resolution data was charge corrected to the reference F 1s signal at 688.9 eV.[12]

X-ray absorption (XAS) measurements were performed at RT at the Pd K-edge in the BL18 beamline at the Diamond Synchrotron Light (experiment number: sp17198). X-ray absorption near edge structure (XANES) and extended x-ray absorption fine structure (EXAFS) spectra of a Pd foil and PdO standards were measured and the energy calibrated by aligning the respective absorption edges. The data were calibrated and normalised by a linear pre-edge subtraction using ATHENA software.[13]

Micro Raman spectroscopy was performed using a Horiba Jobin Yvon LabRAM HR Raman spectrometer. Spectra were acquired using a 785 nm laser (at 24 mW power), a 100x objective lens and a 300 µm confocal pinhole. To simultaneously scan a range of Raman shifts and control the spectral resolution, either a 600 or 1800 lines mm^{-1} rotatable diffraction grating along a path length of 800 mm was employed. Spectra were acquired using a Synapse CCD detector (1024 pixels) thermoelectrically cooled to −60 °C. Before spectra collection, the instrument was calibrated using the zero-order line and a standard Si(100) reference band at 520.7 cm^{-1}. For single point measurements, spectra were acquired over the range 125-1700 cm^{-1} with an acquisition time of 30-120 seconds and 4 accumulations to automatically remove the spikes due to cosmic rays and improve the signal to noise ratio. The spectral resolution is better than 0.8 and 0.2 cm^{-1} for the 600 and 1800 lines mm^{-1} gratings, respectively. All spectra were baseline corrected using a third order polynomial subtraction. To ascertain the relative isomer fraction, spectra were fit within the range 375-430 cm^{-1} with two bands corresponding to the trans (398 cm^{-1}) and cis (407 cm^{-1}) isomers (mixed Gaussian-Lorenzian peak shapes in a fixed 50:50 ratio, fixed 12 cm^{-1} FWHM, max peak shift of 3 cm^{-1}). The peak intensities (as areas) were extracted and the fraction of isomers calculated using the following formulae: $f_{trans} = I_{trans} / (I_{trans} + I_{cis})$ and $f_{cis} = I_{cis} / (I_{trans} + I_{cis})$.

Density functional theory calculations were performed using version 4.1.0 of the Orca Program package.[14] The *cis* and *trans* conformers of [NTf_2] were constructed and combined with both a 1 and 13 Pd atom cluster. [C_4C_1Im][NTf_2] molecules were placed closed to the different facets of the cluster. The structures were optimised, with the lowest energy *cis* and *trans* structures seen in Supplementary Figure 28. The B3LYP functional was used with a dispersion correction[15,16] for all calculations. The def2-TZVP basis set was used along with the relevant auxiliary basis sets for RIJCOSX.[17-19] The Grid5, NoFinalGrid and TightSCF options were used in all cases. As the wave function proved difficult to converge a number of options to aid were tried, such as changing the reset frequency for DIIS steps and also using in some cases the KDIIS algorithm.

General procedure of catalytic experiments

All oxygen and moisture sensitive operations were carried out under argon atmosphere using glove box, standard vacuum-line and Schlenk techniques. Solvents were purchased from Sigma-Aldrich as HPLC grade and dried by means of an Inert Puresolv MD purification system. All reagents were purchased from Sigma Aldrich and purified when required by literature procedures.

^1H and ^{13}C Nuclear Magnetic Resonance (NMR) spectra were recorded on a Bruker DPX300 300 MHz, a DPX400 400 MHz and a DPX500 500 MHz nuclear magnetic resonance spectrometers. Chemical shifts of ^1H and ^{13}C NMR are reported in ppm, the solvent was used as internal standard. Signals are quoted as s (singlet), d (doublet), dd (doublet of doublets), m (multiplet), q (quartet), quint (quintet) sxt (sextet) hept (heptet), dt (doublet of triplets), and td (triplet of doublets) in ^1H NMR spectroscopy.

All reactions were prepared in a glovebox under inert atmosphere using a 50 mL Schlenk flask equipped with a magnetic stir bar. EDA (1 mmol) was added into a solution containing the Pd catalyst (0.5 mol% Pd) and styrene (5 mmol) in CH_2Cl_2 (5/10 mL). The Schlenk flask was sealed with a septum cap and the reaction mixture was removed from the glovebox. After 24 h of stirring at R.T., the mixture was filtered through silica gel and volatiles were removed *in vacuo*. Products have been previously described and their identification was straightforward from comparison with reported data.[20] Substrate conversion, and selectivity and yield of cyclopropane product were determined by ^1H NMR spectroscopy by the use of 1,2-dibromoethane (0.25 mmol) as an internal standard.

In the optimisation of the reaction conditions all experiments were performed as mentioned above for the general catalytic reaction, varying the catalyst, the amount of catalyst (0.5/1 mol%), equivalents of styrene and volume of solvent (5/10 mL of CH_2Cl_2). For slow addition reactions, a solution of EDA (1 mmol) in CH_2Cl_2 (5 mL) was added into a Schlenk flask containing the catalyst (0.5 mol %Pd). Then, a solution of styrene in CH_2Cl_2 was added dropwise over 24 h at R.T. with the aid of a syringe pump. Once the addition was completed, the mixture was filtered through silica gel and

volatiles were removed *in vacuo*. Substrate conversion, and selectivity and yield of cyclopropane product were determined by ^{1}H NMR spectroscopy by the use of 1,2-dibromoethane as internal standard (0.25 mmol).

For the kinetic studies, gas absorption curves were measured with a house made pressure transducer connected to a 5512 Fischer porter reactor and recorded with an E725 RDS transducer indicator. For digitisation RealTeK software was used. Gas absorption curves were processed using in-house software, programmed with LabView programming platform, and Origin software.
In a typical experiment the desired amount of catalyst is added to the reactor vessel in a glove box, and consequently diluted with $[C_4C_1Im][NTf_2]$ so that the reaction contained 500 mg $[C_4C_1Im][NTf_2]$ in total. Afterwards, styrene (5 mmol) and DCM (5 mL) are added the reactor sealed and consequently heated to 35°C under vigorous stirring. Upon reaching the desired temperature, EDA (1 mmol) is injected to the reactor and the gas evolution is measured as described above. The reaction mixture received from these experiments were treated as described previously and analysed *via* ^{1}H NMR in $CDCl_3$ with 1,2-dibromoethane as an external standard.

For the mercury poisoning tests[21], EDA (1 mmol) was added into a solution containing the catalyst (0.5 mol% Pd), styrene (5 mmol) and mercury (400 eq. *vs* Pd) in CH_2Cl_2 (10 mL). After 24 h stirring at R.T., the mixture was filtered through silica gel and volatiles were removed *in vacuo*. Substrate conversion, and selectivity and yield of cyclopropane product were determined by ^{1}H NMR spectroscopy by the use of 1,2-dibromoethane as internal standard (0.25 mmol).

For the DCT-tests, 1 mg or 2 mg of DCT (4.9 µmol, 0.5 mol%, respectively 8.8 µmol, 1 mol%) and the corresponding Pd-catalyst (0.5 mol%) were mixed in CH_2Cl_2 (5 mL) and stirred for 2h at R.T. Then, EDA (1 mmol) and styrene (5 mmol) were added and the reaction was stirred for 24 h at R.T. Finally, the reaction mixture is filtered, volatiles removed under reduced pressure and 1,2-dibromoethane (0.25 mmol) added as internal standard.

For the filtration-tests, the corresponding Pd catalyst is dissolved in CH_2Cl_2 (10 mL) and passed through a small plug of celite (1 g) under inert atmosphere. Afterwards, the plug is washed with CH_2Cl_2 (30 mL) and all volatiles are removed under vacuum. The catalytic experiments are conducted as already described.

For the recycling experiments all reactions were prepared in a glovebox under inert atmosphere using a 100 mL Schlenk flask equipped with a magnetic stir bar. EDA (1 mmol) was added into a solution containing the Pd catalyst (0.5 mol% Pd) and styrene (5 mmol) in CH_2Cl_2 (5mL). The Schlenk flask was sealed with a septum cap. After 24 h of stirring at R.T., all volatiles are removed under vacuum and the catalytic phase washed (3x15 mL) with *n*-hexane. The insoluble catalytic phase is dried under vacuum and used for consecutive cycles. The combined organic phases are reduced

under vacuum, and 1,2-dibromoethane (0.25 mmol) is added as an internal standard to determine the conversion *via* NMR. The product phase is afterwards digested with aqua regia for 24h, diluted with 5% HCl and the metal content determined *via* ICP-OES.

The substrate scope experiments were performed as described above for the general catalytic reaction by the use of the corresponding olefin (5 mmol) and Pd@[C_4C_1Im][NTf_2] as catalyst (0.5 mol% Pd). All the products have been previously described and identified by comparison with reported data.[20,22-24]

Supplementary References

1 Venkatesan, R. *et al.* Palladium nanoparticle catalysts in ionic liquids: synthesis, characterisation and selective partial hydrogenation of alkynes to Z-alkenes. *J. Mater. Chem.* **21**, 3030-3036, (2011).

2 Paschoal, V. H., Faria, L. F. O. & Ribeiro, M. C. C. Vibrational Spectroscopy of Ionic Liquids. *Chem. Rev.* **117**, 7053-7112, (2017).

3 Bonhôte, P., Dias, A.-P., Papageorgiou, N., Kalyanasundaram, K. & Grätzel, M. Hydrophobic, Highly Conductive Ambient-Temperature Molten Salts. *Inorg. Chem.* **35**, 1168-1178, (1996).

4 Crowhurst, L., Mawdsley, P. R., Perez-Arlandis, J. M., Salter, P. A. & Welton, T. Solvent–solute interactions in ionic liquids. *Phys. Chem. Chem. Phys* **5**, 2790-2794, (2003).

5 Leu, M. K. *et al.* On the real catalytically active species for CO_2 fixation into cyclic carbonates under near ambient conditions: Dissociation equilibrium of [BMIm][$Fe(NO)_2Cl_2$] dependant on reaction temperature. *Appl. Catal. B* **245**, 240-250, (2019).

6 Huddleston, J. G. *et al.* Characterization and comparison of hydrophilic and hydrophobic room temperature ionic liquids incorporating the imidazolium cation. *Green Chem.* **3**, 156-164, (2001).

7 Helmchen, G. Dibenzo[a,e]cyclooctene: Multigram Synthesis of a Bidentate Ligand. *Org. Synth.* **89**, 55-65, (2012).

8 Neate, P. G. N., Greenhalgh, M. D., Brennessel, W. W., Thomas, S. P. & Neidig, M. L. Mechanism of the Bis(imino)pyridine-Iron-Catalyzed Hydromagnesiation of Styrene Derivatives. *J. Am. Chem. Soc.* **141**, 10099-10108, (2019).

9 Nordlund, K. Molecular dynamics simulation of ion ranges in the 1–100 keV energy range. *Comput. Mater. Sci.* **3**, 448-456, (1995).

10 Berendsen, H. J. C., Postma, J. P. M., van Gunsteren, W. F., DiNola, A. & Haak, J. R. Molecular dynamics with coupling to an external bath. *J. Chem. Phys* **81**, 3684-3690, (1984).

11 Ziegler, J. F. & Biersack, J. P. in *Treatise on Heavy-Ion Science: Volume 6: Astrophysics, Chemistry, and Condensed Matter* (ed D. Allan Bromley) 93-129 (Springer US, 1985).

12 Santos, A. R., Blundell, R. K. & Licence, P. XPS of guanidinium ionic liquids: a comparison of charge distribution in nitrogenous cations. *Phys. Chem. Chem. Phys* **17**, 11839-11847, (2015).

13 Sun, X. *et al.* Facile synthesis of precious-metal single-site catalysts using organic solvents. *Nat. Chem.*, (2020).

14 Neese, F. Software update: the ORCA program system, version 4.0. *WIREs Comput. Mol. Sci.* **8**, e1327, (2018).

15 Grimme, S., Ehrlich, S. & Goerigk, L. Effect of the damping function in dispersion corrected density functional theory. *J. Comput. Chem.* **32**, 1456-1465, (2011).

16 Grimme, S., Antony, J., Ehrlich, S. & Krieg, H. A consistent and accurate ab initio parametrization of density functional dispersion correction (DFT-D) for the 94 elements H-Pu. *J. Chem. Phys* **132**, 154104, (2010).

17 Weigend, F. & Ahlrichs, R. Balanced basis sets of split valence, triple zeta valence and quadruple zeta valence quality for H to Rn: Design and assessment of accuracy. *Phys. Chem. Chem. Phys* **7**, 3297-3305, (2005).

18 Weigend, F. Accurate Coulomb-fitting basis sets for H to Rn. *Phys. Chem. Chem. Phys* **8**, 1057-1065, (2006).

19 Hellweg, A., Hättig, C., Höfener, S. & Klopper, W. Optimized accurate auxiliary basis sets for RI-MP2 and RI-CC_2 calculations for the atoms Rb to Rn. *Theor. Chem. Acc.* **117**, 587-597, doi:10.1007/s00214-007-0250-5 (2007).

20 Cho, D.-J. *et al.* Chiral C_2-symmetric bisferrocenyldiamines as ligands for transition metal catalyzed asymmetric cyclopropanation and aziridination. *Tetrahedron: Asymmetry* **10**, 3833-3848, (1999).

21 Widegren, J. A. & Finke, R. G. A review of the problem of distinguishing true homogeneous catalysis from soluble or other metal-particle heterogeneous catalysis under reducing conditions. *J. Mol. Catal. A Chem.* **198**, 317-341, (2003).

22 Martín, C., Molina, F., Alvarez, E. & Belderrain, T. R. Stable N-Heterocyclic Carbene (NHC)–Palladium(0) Complexes as Active Catalysts for Olefin Cyclopropanation Reactions with Ethyl Diazoacetate. *Chem. Eur. J* **17**, 14885-14895, (2011).

23 Doyle, M. P. & Hu, W. Selectivity in Reactions of Allyl Diazoacetates as a Function of Catalyst and Ring Size from γ-Lactones to Macrocyclic Lactones. *J. Org. Chem.* **65**, 8839-8847, (2000).

24 Wolf, J. R., Hamaker, C. G., Djukic, J.-P., Kodadek, T. & Woo, L. K. Shape and stereoselective cyclopropanation of alkenes catalyzed by iron porphyrins. *J. Am. Chem. Soc.* **117**, 9194-9199, (1995).
